# Mechanical stress confers nuclear and functional changes in derived leukemia cells from persistent confined migration

**DOI:** 10.1007/s00018-023-04968-5

**Published:** 2023-10-06

**Authors:** Ana de Lope-Planelles, Raquel González-Novo, Elena Madrazo, Gracia Peralta-Carrero, María Pilar Cruz Rodríguez, Héctor Zamora-Carreras, Verónica Torrano, Horacio López-Menéndez, Pedro Roda-Navarro, Francisco Monroy, Javier Redondo-Muñoz

**Affiliations:** 1 Department of Molecular Medicine, Centro de Investigaciones Biológicas Margarita Salas (CIB Margarita Salas-CSIC), Madrid, Spain; 2 Department of Immunology, School of Medicine, University Complutense de Madrid and 12 de Octubre Health Research Institute (Imas12) Madrid, Madrid, Spain; 3 Department of Biochemistry and Molecular Biology, University of the Basque Country, Leioa, Spain; 4 Department of Physical Chemistry, Complutense University, Madrid, Spain; 5 Translational Biophysics, Hospital Doce de Octubre Health Research Institute (imas12), Madrid, Spain

**Keywords:** Cell migration, Nuclear deformability, Mechanobiology, Lamin, DNA damage

## Abstract

Nuclear deformability plays a critical role in cell migration. During this process, the remodeling of internal components of the nucleus has a direct impact on DNA damage and cell behavior; however, how persistent migration promotes nuclear changes leading to phenotypical and functional consequences remains poorly understood. Here, we described that the persistent migration through physical barriers was sufficient to promote permanent modifications in migratory-altered cells. We found that derived cells from confined migration showed changes in lamin B1 localization, cell morphology and transcription. Further analysis confirmed that migratory-altered cells showed functional differences in DNA repair, cell response to chemotherapy and cell migration in vivo homing experiments. Experimental modulation of actin polymerization affected the redistribution of lamin B1, and the basal levels of DNA damage in migratory-altered cells. Finally, since major nuclear changes were present in migratory-altered cells, we applied a multidisciplinary biochemical and biophysical approach to identify that confined conditions promoted a different biomechanical response of the nucleus in migratory-altered cells. Our observations suggest that mechanical compression during persistent cell migration has a role in stable nuclear and genomic alterations that might handle the genetic instability and cellular heterogeneity in aging diseases and cancer.

**Supplementary Information:**

The online version contains supplementary material available at 10.1007/s00018-023-04968-5.

## Introduction

Cell migration is a fundamental process involved in physiological and pathological conditions such as development, immune response, inflammation, and cancer invasion [1]. Migrating cells have to cross through different tissue environments, including the extracellular matrix and endothelial barriers, which provide multiple biochemical and mechanobiological signals for an active and effective cell migration [2, 3]. The nucleus is a highly dynamic organelle composed of the nuclear envelope, the lamina network, and the chromatin structure [4], which contribute and define the nuclear deformability to allow cell migration across physical barriers [5]. It has been reported that the nuclear changes induced by external stimuli have to be transmitted through the cytoskeleton and other intracellular pathways [6, 7]; and this nuclear deformation induced by mechanical stress regulates chromatin changes, DNA damage response, and cell stemness [8–12]. Likely, several human pathologies are associated with defects in the response of the nucleus to mechanical stimuli, including progeria, cardiomyopathies, and cancer [13, 14]. However, it is not yet clear how persistent migration controls stable and consistent nuclear changes that might contribute to genomic alterations with functional consequences in homeostasis and pathological situations.

In our study, we investigated two cell migration settings, one-round migration (ORM) cells, and migratory-altered (MA) cells, which were derived from passage through three sequential rounds of confined migration and kept consistent nuclear changes. We demonstrated that cell migration across restrictive pore size triggered morphological and phenotypical changes, including an aberrant distribution of lamin B1. Furthermore, these changes in MA cells were stable in the absence of further mechanical stress. Our results indicate that MA cells altered their transcriptional profile, their basal levels of DNA damage and a range of mechanical and functional responses, including their in vivo migration and their sensitivity to chemotherapy. We combined DNA sensitivity assays, confined compression, atomic force microscopy and optical tweezers, to demonstrate that the chromatin conformation and the biophysical response of the nuclei from MA cells showed a different behavior than control cells, suggesting a mechanical adaptation of the chromatin and the nucleus induced by persistent migration. Noticeably, we observed that latrunculin treatment rescued the normal phenotype and localization of lamin B1 at the nuclear periphery and chromatin compaction, indicating that actin polymerization might influence these changes. Overall, our findings show that nuclear deformability induced by cell migration might contribute to genomic alterations that could lead to functional changes, such as tumor progression and heterogeneity of cancer cells.

## Results

### Mechanical constriction promotes morphological changes in the nucleus of migrating cells.

**Fig. 1:**
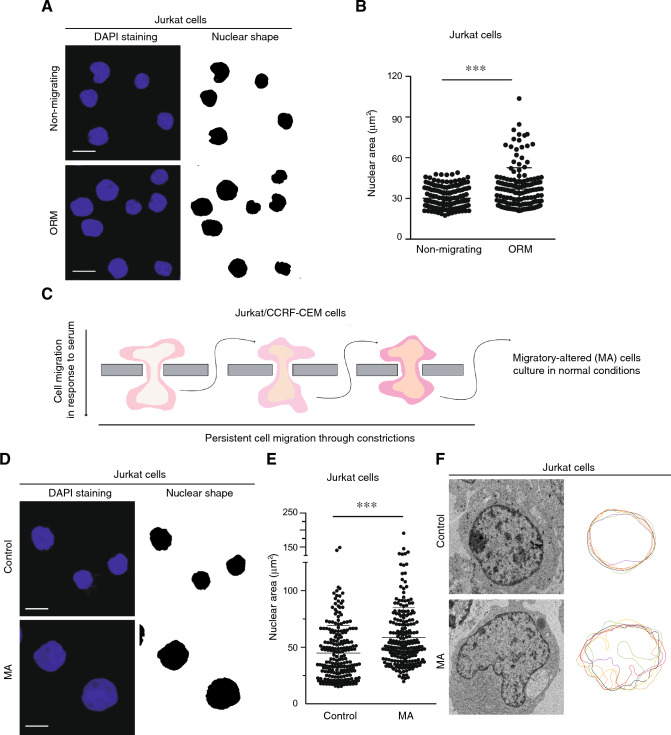
Cell migration through constrictions governs nuclear changes in leukemia cells. **A** Jurkat cells were allowed to migrate across 3 μm transwell inserts for 24 h. Non-migrating and one-round migrated (ORM) cells were collected from the upper and bottom chambers, respectively, sedimented on poly-L-lysine coated coverslips, fixed and stained with DAPI (blue) for their analysis by confocal microscopy. Right panels indicate in black the area of the nuclei. Bar 10 μm. **B** Graph shows changes in the nuclear area of Jurkat cells upon one-round of migration through constrictions. Mean n = 157 cells ± SD (3 replicates). **C** Jurkat and CCRF-CEM cells were forced to migrate three repeated rounds through 3 μm transwell inserts. Then, migrated cells were collected as migratory-altered (MA) cells, expanded and kept in culture conditions. **D** Control and MA Jurkat cells were sedimented on poly-L-lysine coated coverslips, fixed and stained with DAPI. Right panels indicate in black the area of the nuclei. Bar 10 μm. **E** Graph shows changes in the nuclear area of control and MA cells. Mean n = 216 cells ± SD (5 replicates). **F** Control and MA Jurkat cells were collected and processed for thin section electron microscopy to visualize the nuclear morphology. Plot shows changes in the nuclear circularity of control and MA Jurkat cells (n = 6 representative cells). (***) P < 0.001

To determine how the mechanical stress induced by confined migration might alter the nuclear morphology of migrating cells, we first seeded leukemia cells on rigid filter membranes and allowed their migration (one-round migration, ORM) through 3 μm pores. It has been previously reported that for leukocytes 3 μm is the pore size that requires an active nuclear deformability during cell migration, whilst 5 μm pore size showed a much less restrictive space for lymphocyte migration [15, 16]. After 24 h, we collected these cells and compared them with their non-migrating counterparts to characterize how cell migration through physical barriers might influence the nucleus of leukemia cells. We observed that ORM cells increased their nuclear area in comparison to non-migrating cells (Fig. 1A, B, Supplementary Fig. S1A, B). It has been shown that changes in the volume of isolated nuclei can be determined by flow cytometry [17], and we verified that isolated nuclei from ORM cells showed bigger volume than nuclei from non-migrating cells (Supplementary Fig. S1C). To investigate the long-term consequences of a consistent migratory stimulus, we derived stable MA cells by allowing cells to undergo three sequential rounds of confined migration. Cells that migrated successfully after the three rounds were collected and kept in suspension without any further mechanical stimuli (Fig. 1C). It has been reported that tumor cells migrating across numerous constrictions might promote stable changes in the chromatin disposition and cellular morphology [18]. In the attempt to identify whether repeated migratory stress might induce changes in the nuclear morphology of MA cells, we visualized their nuclei and confirmed an increment in the nuclear area of MA cells compared to control cells (Fig. 1D, E, Supplementary Fig. S1D, E). These results were in line with our findings that ORM cells presented bigger nuclei compared to control cells. Having observed differences in the size of the nuclei, we next focused on the nuclear morphology of MA cells. Transmission electron microscopy (TEM) images revealed a remarkable aberrant morphology of the nucleus of MA cells compared to those from control cells (Fig. 1F, and Supplementary Fig. S1F); although we did not observe differences in the nuclear morphology of ORM cells (Supplementary Fig. S1G, H). Together, these data indicate that cell squeezing during migration induces changes in the nuclear size of moving cells, whilst altering nuclear morphology might be characteristic of cells undergoing persistent migration.

### Persistent migration alters permanently the lamin B1 distribution within the nucleus

**Fig. 2:**
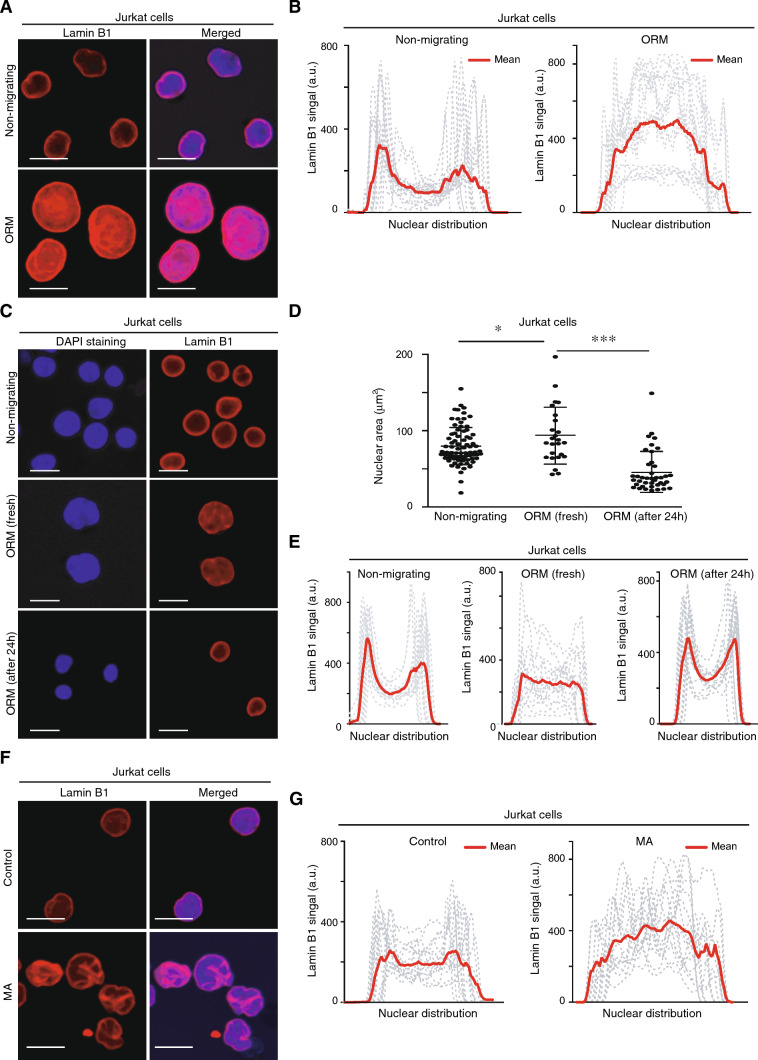
Confined migration alters the lamin B1 distribution of moving cells. **A** Non-migrating and ORM Jurkat cells were seeded on poly-L-lysine-coated glasses and stained with DAPI (blue) and anti-lamin B1 antibody (red) for their analysis by confocal microscopy. Bar 10 μm. **B** Line plots show the signal profile of lamin B1 from 15 representative nuclei. Red line indicates the mean intensity of the profiles analyzed. **C** Control, fresh ORM, and ORM Jurkat cells collected and cultured in suspension for an additional 24 h were seeded on poly-L-lysine-coated glasses and analyzed by confocal microscopy. Bar 10 μm. **D** Graph shows changes in the nuclear area of the cells from (C). Mean n = 25–92 cells ± SD (2 independent replicates). **E** Line plots show the signal profile of lamin B1 from 15 representative nuclei of the cells from (**C**). **F** Control and MA Jurkat cells were seeded on poly-L-lysine-coated glasses and analyzed by confocal microscopy. Bar 10 μm. **G** Line plots show the signal profile of lamin B1 from 15 representative nuclei of control and MA Jurkat cells. The red line indicates the mean intensity of the profiles analyzed. (*) P < 0.05, (***) P < 0.001

In contrast to adherent tumor cells, which express high levels of lamin A that might be altered upon mechanical compression [18, 19], the major component of the nuclear lamina in leukemia cells is B-type lamin [20]. We next sought to elucidate whether confined migration promoted alterations in the lamina network and found that constricted migration altered the distribution of lamin B1 in ORM cells (Fig. 2A, and Supplementary Fig. S2A). While non-migrating cells showed two characteristic peaks for lamin B1 signal in the transversal section of the nucleus (corresponding to the nuclear periphery), ORM cells presented an aberrant distribution of lamin B1 (Fig. 2B, Supplementary Fig. S2B, and Supplementary Movies S1, S2). Temporal changes in internal components might critically regulate the morphology of the nucleus in moving cells; therefore, changes in the nuclear volume and lamin B1 redistribution of ORM cells would be recovered once the migratory effect disappears. We demonstrated that ORM cells cultured in suspension for an additional 24 h reduced their nuclear size to levels comparable to those from non-migrating cells (Fig. 2C, D, Supplementary Fig. S2C, and S2D). Furthermore, these cells also recovered the normal localization of lamin B1 at the nuclear periphery (Fig. 2E, and Supplementary Fig. S2E). Notably, these results indicated that nuclear changes observed in ORM cells were temporal and remained only for a short period of time. On the contrary, MA cells showed stable differences in the morphology and size of the nucleus compared to control cells (see above, Fig. 1); and we found that MA cells also presented an aberrant distribution of lamin B1 (Fig. 2F, G, Supplementary Fig. S3A, B, and Supplementary Movies S3, S4). Aberrant nuclear morphology might be characterized by the accumulation of nuclear lobes enriched in nuclear envelope proteins. Therefore, we stained ORM cells with emerin (a nuclear envelope marker) and found that the nucleus of ORM cells also presented a remarkable signal of emerin in the nuclear invaginations, (Supplementary Fig. S3C, and Movie S5). Then, we stained MA cells with emerin and an additional nuclear envelope protein (sun2) and found a similar distribution of both proteins, suggesting that the nucleus of MA cells was displayed in multiple nuclear lobes (Supplementary Fig. S3D, and Movies S6, S7). Altogether, these results indicate that confined migration promotes lamin B1 distribution in nuclear lobes, which is stable within time after persistent repeated migration, whilst transient migration (ORM) shows only a temporary effect on the nucleus.

### Persistent migration induces enduring changes in the transcriptional profile of migrating cells

**Fig. 3:**
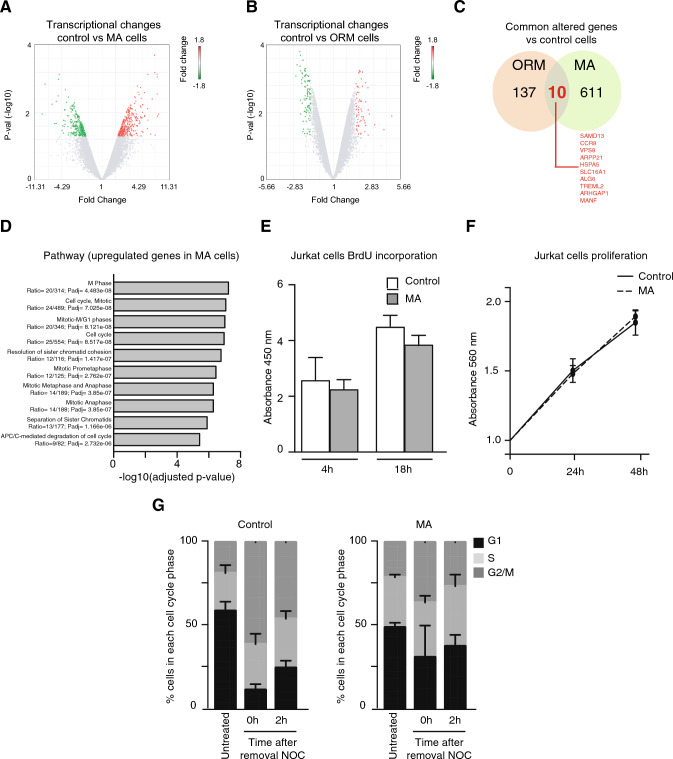
Persistent migration induces changes in the transcriptional profile of migrating cells. **A** mRNA from control and MA Jurkat cells was isolated and the number of differentially expressed genes was analyzed by microarray. Volcano plot shows significant changes of gene transcripts in control and MA cells. **B** Control and ORM Jurkat cells were lysed, and the number of differentially expressed genes was analyzed by microarray. Volcano plot shows significant changes of gene transcripts in control and ORM cells. **C** Venn diagram representation of common transcriptional changes (10 transcripts, labeled in red) between ORM and MA cells compared to control cells. **D** Pathway enrichment analysis based on differentially upregulated genes in MA Jurkat cells. **E** Control and MA Jurkat cells were incubated with BrdU for 4 and 18 h. Then, cells were fixed and BrdU incorporation quantified. Mean n = 6 replicates ± SD. **F** Control (dark line) and MA (dashed line) Jurkat cells were cultured at indicated times and cell proliferation was quantified by MTT assay. Mean n = 3 replicates ± SD. **G** Control and MA cells were cultured in the presence or not of nocodazole (100 ng/ml) for 16 h. Then, cells were washed and the cell cycle was determined at indicated times. Bar graph shows the cell cycle profile analysis of control and MA cells following release from nocodazole inhibition. Mean n = 3 replicates ± SD

It has been previously described that nuclear shape regulates the transcriptional activity of cells [21]. As MA cells showed a different and sustained phenotype compared to control cells, we hypothesized that persistent cell migration might produce enduring changes in the transcriptional landscape of MA cells. We evaluated the expression levels of transcripts of control or MA cells and found a significant differential expression of 1.8-fold change in 621 genes (283 up- and 338 down-regulated) in MA cells compared with control cells (Fig. 3A, Supplementary Fig. S4A, and Supplementary Table S1). We next extended our observation to KEGG (Kyoto Encyclopedia of Genes and Genomes) and GO (Gene Ontology) enrichment analyses, which revealed that most of the altered genes were connected to cell division, survival, DNA damage checkpoints, and protein modification (Supplementary Fig. S4B). We validated our microarray analysis by confirming the mRNA downregulation of two genes related to leukemia (*EZH2* and *JAK2*), as well as the expression levels of 5 randomly selected proteins (Supplementary Fig. S4C–E). Once validated these observations for MA cells, we analyzed whether one single round of cell migration might be sufficient to induce transcriptional changes in moving cells, and found that ORM cells altered only the transcription of 147 genes (90 up- and 57 down-regulated) compared to control cells (Fig. 3B, and Supplementary Table S2). When we compared the transcriptional changes of ORM and MA cells with respect to controls, we found that both cell types only shared 10 altered genes in common (Fig. 3C). Previous reports showed that confined conditions regulate cell proliferation and cell cycle progression [22, 23]; and our pathway analysis indicated an upregulation of genes related to G2/M progression, mitotic spindle, and chromosome segregation in MA cells (Fig. 3D, and Supplementary Table S3). However, we observed no differences in the proliferative properties of MA cells compared to control cells (Fig. 3E, F and Supplementary Fig. S4E, F), nor in the cell cycle progression of MA cells (Supplementary Fig. S4G). To further determine whether cell cycle progression might be affected in MA cells, we treated control or MA cells with nocodazole (a chemical inhibitor that disrupts microtubules and arrests cells before mitosis) and followed cell cycle progression. We observed that MA cells were less sensitive to nocodaloze arrest (Fig. 3G), suggesting that under external stimuli, or specific conditions the cell proliferation of MA cells might be affected. Overall, our results indicate that confined migration through constrictions might contribute to alter the transcriptional profile of moving cells.

### Migrating cells through confined conditions show altered basal levels of DNA damage markers and resistance to apoptosis

**Fig. 4:**
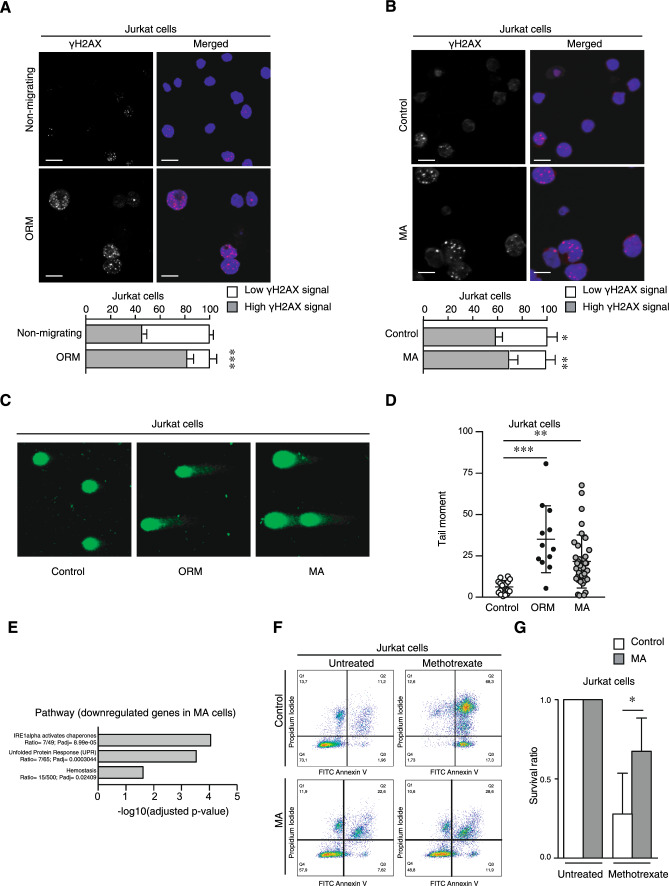
Confined migration leads to aberrant DNA damage response of moving cells. **A** Non-migrating and ORM Jurkat cells were seeded on poly-L-lysine-coated glasses and stained with DAPI (blue) and anti-γH2AX antibody (red) for their analysis by confocal microscopy. Bar 10 μm. Graph shows the percentage of cells with more than 2 visible foci for γH2AX (dark grey, as high γH2AX signal). Mean n = 116–118 ± SD (3 replicates). **B** Control and MA Jurkat cells were seeded on poly-L-lysine-coated glasses, stained, and analyzed by confocal microscopy. Bar 10 μm. Graph shows the percentage of control and MA Jurkat cells with more than 2 visible foci for γH2AX. Mean n = 28–35 cells ± SD (2 replicates). **C** Control, ORM, and MA Jurkat cells were embedded in agarose and lysed. Alkaline comet assay by electrophoresis was performed to visualize the DNA by fluorescence microscopy. **D** Graph shows the tail moment analysis of the comet assay in (**C**). Mean n = 32–60 cells ± SD (3 replicates). **E** Pathway enrichment analysis based on differentially downregulated genes in MA Jurkat cells. **F** Control and MA Jurkat cells were cultured in the presence or not of methotrexate (1 μM) for 24 h. Then, cells were collected and stained with annexin V-FITC and propidium iodide for their flow cytometry analysis. **(G)** Graph shows the survival ratio of control and MA Jurkat cells upon normalization to their untreated conditions. Mean n = 3 ± SD. (*) P < 0.05, (**) P < 0.001, (***) P < 0.001

To further elucidate the functional consequences of nuclear changes induced by persistent cell migration, we explored how MA cells might present differences in their survival and response to apoptosis. Cell migration is also known to favor DNA damage upon extreme nuclear deformability [24, 25], and we found that most of the non-migrating cells showed low levels of γH2AX (a well-known DNA damage marker), in contrast to fresh ORM cells (Fig. 4A). Likewise, we studied the levels of γH2AX and phospho-ATM (another DNA damage marker) in MA cells and found higher levels of both proteins in MA cells compared to control cells (Fig. 4B, and Supplementary Fig. S5A–C), indicating that cells undergoing transient and persistent migration increased the basal levels of DNA damage. To further confirm this hypothesis, we performed a comet assay of control, ORM, and MA cells to measure the basal DNA damage level. We quantified the length and intensity of the comet tail and found that comet descriptors were higher for ORM and MA cells compared to controls (Fig. 4C, D). These observations prompted the hypothesis that the mechanical stress during cell migration would presumably have an impact on how leukemia cells respond to therapy. Interestingly, our pathway analysis showed that MA cells might downregulate genes involved in endoplasmic reticulum stress and unfolded protein response (Fig. 4E and Supplementary Table S4), which might be relevant for drug resistance in cancer [26]. To assess how persistent migration might affect cell viability, we treated control and MA cells with methotrexate, a DNA biosynthesis inhibitor used in clinics against multiple human pathologies [27]. Notably, we found that MA cells showed higher resistance to apoptosis than control cells (Fig. 4F, G). A similar tendency was observed using lower or higher concentrations of methotrexate (Supplementary Fig. S5D). To further confirm these results, we treated cells with bleomycin to induce DNA damage and found that control cells were more sensitive to DNA damage and presented a bigger tail moment than ORM and MA cells, consistent with our previous observation using methotrexate (Supplementary Fig. S5E, F). Overall, these observations suggest that persistent migration would facilitate the resistance to apoptosis and the adaptation to conventional therapies of invasive moving cells.

### Persistent migration promotes changes in the chemotactic response of moving cells and their capacity to infiltrate tissues in vivo

**Fig. 5:**
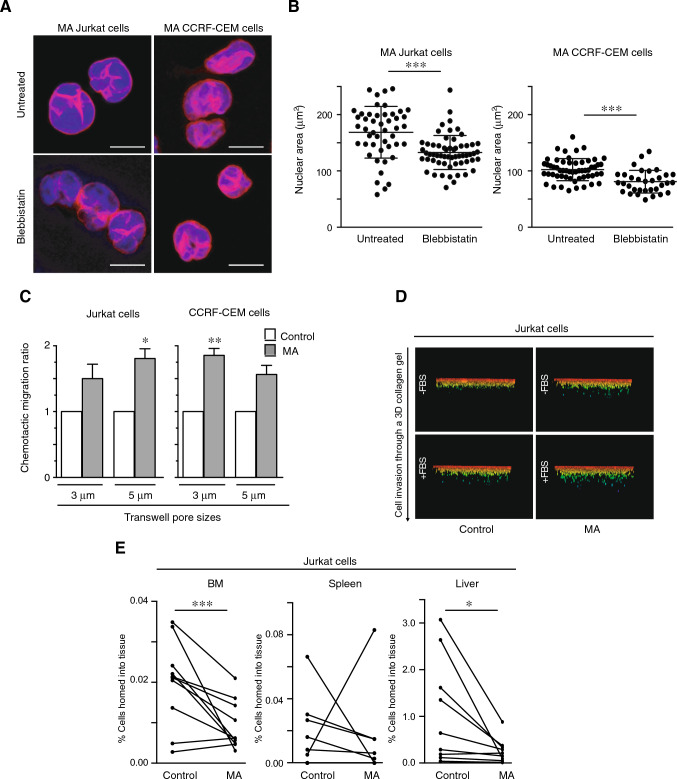
Confined migration has a dual impact on the chemotactic response and in vivo invasiveness of moving cells. **A** MA Jurkat cells were cultured in the presence or absence of blebbistatin (1 μg/mL) for 1 h at 37 ºC. Then, cells were seeded on poly-L-lysine coated coverslips, fixed and analyzed by confocal microscopy. Bar 10 μm. **B** Graph shows the nuclear area from cells in (**A**). Mean n = 132–158 isolated nuclei ± SD. **C** Control and MA Jurkat and CCRF-CEM cells were seeded on the top of Transwell chambers and allowed to migrate in response to serum (FBS). Cells were collected from the bottom chamber after 24 h and quantified. Mean n = 3 replicates ± SD. **D** Control and MA Jurkat cells were seeded on the top of a collagen matrix and allowed to penetrate into the collagen in response to serum (FBS, fetal bovine serum) for 24 h. Cells were fixed, stained with propidium iodide and serial confocal sections were captured. Images show the cell penetrability into the collagen. **E** Control (Cell Tracker Far Red +) and MA (CFSE +) Jurkat cells were mixed and injected into the tail vein of 10 NSG mice. After 24 h, labeled cells in spleen, liver and bone marrow were determined by flow cytometry. Graph shows the percentage of control and the MA cells analyzed in each animal. Mean n = 10. (*) P < 0.05,(**) P < 0.001, (***) P < 0.001

Given that actomyosin contractility controls the nuclear size and cell migration [28, 29], we first tested whether the levels of phospho-myosin might be altered in MA cells. We did not observe remarkable differences in the activation of myosin in control or MA cells (Suppl Fig. S6A). Moreover, we treated MA cells with blebbistatin (a myosin contractility inhibitor) and evaluated whether myosin inhibition might alter the aberrant lamin B1 redistribution of MA cells. Our results indicate that lamin B1 remained in its aberrant redistribution in MA cells treated with blebbistatin (Fig. 5A), whilst the nuclear area was reduced (Fig. 5B). As confined migration has been linked to invasive phenotypes of cancer cells, we interrogated whether MA cells might acquire a different migratory capacity and found that they showed a higher chemotactic response compared to control cells in vitro (Fig. 5C). We also observed that MA cells overexpressed the subunit β1 of integrin, which is involved in cell adhesion and leukemia infiltration (Supplementary Fig. S6B). Interestingly, MA cells showed a slight increment in their capacity to penetrate into a 3D collagen matrix, even in the absence of FBS (Fig. 5D, and Supplementary Fig. S6C). To advance on these in vitro results and determine the ability of MA cells to infiltrate into the bone marrow and spleen of immunodeficient mice [30], we injected an equal number of control and MA cells labeled with vital dyes into the tail vein of recipient mice and found a reduction of MA cells reaching these tissues related to harboring metastatic leukemia cells (Fig. 5E, and Supplementary Fig. S6D). These results indicate that persistent confined migration alters the distribution of lamin B1 independent of myosin contractility and has a dual impact on the migratory capacity of MA cells, increasing their chemotactic response and reducing their invasiveness in vivo.

### Aberrant lamin B1 redistribution and basal DNA damaged induced by persistent migration are dependent on actin polymerization

**Fig. 6:**
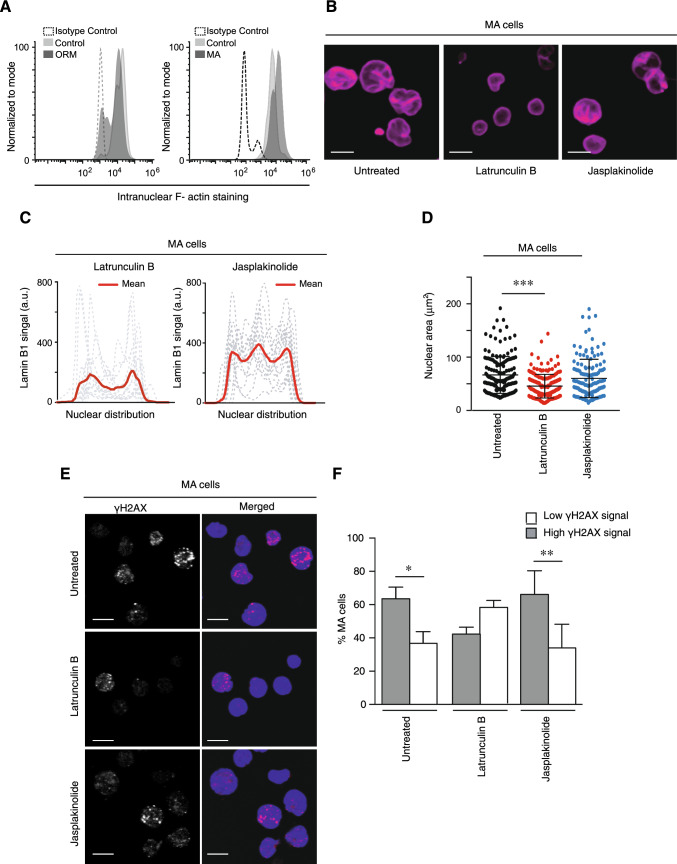
Actin polymerization balance regulates the nuclear changes described for MA cells. **A** Flow cytometry expression of F-actin in the isolated nuclei of control, ORM and MA cells. **B** MA Jurkat cells were cultured in the presence or absence of latrunculin B (1 μg/mL) and jasplakinolide (1 μg/mL) for 1 h at 37ºC. Then, cells were seeded on poly-L-lysine coated coverslips, fixed and analyzed by confocal microscopy. Bar 10 μm. **C** Line plots show the signal profile of lamin B1 from 15 representative nuclei. The red line indicates the mean intensity of the profiles analyzed. **D** Graph shows changes in the nuclear area of MA Jurkat cells under indicated treatments. Mean n = 145–185 nuclei ± SD (3 replicates). **E** Control and MA Jurkat cells were treated or not with latrunculin B or jasplakinolide for 1 h. Cells were fixed, permeabilized and stained with DAPI (blue) and γH2AX (red) for their analysis by confocal microscopy. Bar 10 μm. **F** Graph shows the percentage of cells with more than 2 visible foci for γH2AX from (E). Mean n = 53–82 nuclei ± SD (3 replicates). (*) P < 0.05,(**) P < 0.001, (***) P < 0.001

An additional actor involved in cell migration and nuclear changes is the actin cytoskeleton, which reorganizes around the nucleus regulating the nuclear morphology and chromatin upon mechanical stress [31, 32]. We stained control, ORM and MA cells with phalloidin to determine how cells attached onto 2D surface and whether perinuclear actin might reorganize around or over the nucleus. We did not see any significant difference in perinuclear filaments and the F-actin disposition (Supplementary Fig. S7A); however, we observed differences in the cell spreading of ORM and MA cells compared to control cells (Supplementary S7B). Interestingly, when we analyzed the levels of F-actin in the nucleus, we observed an increment of F-actin in MA cells compared to controls, whilst isolated nuclei from ORM cells showed a slight reduction in the levels of F-actin (Fig. 6A). As we found that lamin B1 redistributed in MA cells, we used specific drugs to depolymerize or stabilize actin filaments and found that latrunculin B (an inhibitor against actin polymerization) treatment was sufficient to rescue the localization of lamin B1 at the nuclear periphery in MA cells (Fig. 6B, C, Supplementary Fig. S7C, D). Likewise, MA cells treated with latrunculin B showed a reduction in the nuclear area, whilst the pharmacological stabilization of polymerized actin with jasplakinolide did not promote any effect (Fig. 6D). In addition to actin polymerization, several kinases trigger lamin B1 redistribution, such as Cdk1 (cyclin-dependent kinase 1) and protein kinase C (PKC) [33]. To determine whether these kinases might affect the phenotype observed in MA cells, we incubated these cells with specific inhibitors and observed that none of them rescued the normal lamin B1 redistribution (Supplementary Fig. S7E). Moreover, we did not see any reduction in the nuclear area of MA cells upon these treatments, and even roscovitine, a Cdk1 inhibitor, seemed to increase it (Supplementary Fig. S7F). Finally, several reports show that actin cytoskeleton is required during DNA damage response [34, 35]. Notably, we found that actin polymerization also affected the levels of γH2AX in MA cells, as latrunculin B treatment reduced the basal levels of γH2AX, whereas jasplakinolide treatment exhibited a more similar phenotype to untreated MA cells (Fig. 6E, F). Taken together, our results indicate that actin polymerization might mediate the nuclear changes induced by persistent cell migration in MA cells.

### Migrating cells through confined conditions alter their global chromatin compaction state

**Fig. 7:**
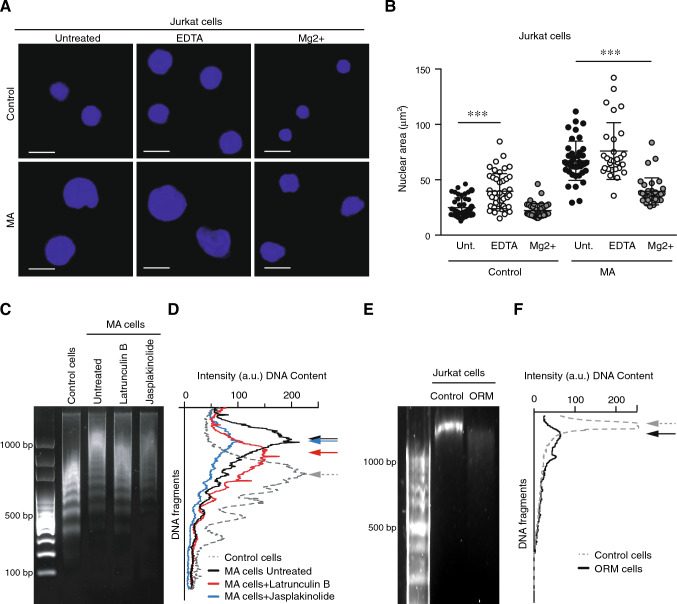
Confined migration promotes changes in the global chromatin configuration of moving cells. **A** Isolated nuclei from control and MA Jurkat cells were seeded on poly-lysine coated glasses. Then, osmotic stress conditions were induced by the addition of EDTA (swelling conditions) or MgCl_2_ (shrinking conditions). Nuclei were fixed, permeabilized and stained with DAPI for their analysis by confocal microscopy. Bar 10 μm. **B** Graph shows the nuclear area quantified from (**C**). Mean n = 29–46 isolated nuclei ± SD (2 replicates). **C** Control and MA Jurkat cells were treated or not with latrunculin B or jasplakinolide for 1 h. Then, cells were collected, and their DNA was digested with DNAse for 15 min and resolved in an agarose gel. **D** Graph shows the nucleosomal releasing profile from control (dashed line) and MA Jurkat cells as in (C). Arrows indicate the maxima DNA peaks in each cell population. **E** Control and ORM Jurkat cells were collected, and their DNA was digested with DNAse for 15 min and resolved in an agarose gel. **F** Graph shows the nucleosomal releasing profile from control (dashed line) and ORM Jurkat. Arrows indicate the maxima DNA peaks in each cell population. (***) P < 0.001

As a first approach to characterize the mechanical behavior of the nucleus, we addressed its response under osmotic stress. The balance of ions regulates the osmotic pressure and the chromatin compaction in the nucleus [36]. We observed that isolated nuclei from MA cells responded better to an increase of the concentration of cations (42% of shrinking compared to 10% of nuclei from control cells), whilst the cation depletion caused by the addition of EDTA promoted higher swelling in isolated nuclei from control cells rather than from MA cells (60% and 30% of increment in the nuclear area, respectively; Fig. 7A, B). To further demonstrate that MA cells might present differences in the chromatin compaction state, we treated MA cells with chaetocin (a histone methyltransferase inhibitor) and found that it increased the nuclear area of MA cells (Supplementary Fig. S8A), suggesting that the chromatin compaction of MA cells was different to control cells and sensitive to histone modifiers activity. We interrogated whether the disposition of lamin B1 might be also affected by osmotic stress. First, we observed that the nuclear isolation process reduced the aberrant lamin B1-stained lobs we described in intact ORM and MA cells (Fig. S8B). We also observed that swelling or shrinking conditions did not alter significantly the localization of lamin B1 (Fig. S8B). Similarly to the response of isolated nuclei from MA cells to osmotic stress, we isolated the nuclei from ORM cells and found that a similar increment of the nuclear area in untreated conditions and a bigger shrinking capacity upon cation addition (Fig. S8C).

The global chromatin compaction might be determined by the genomic sensitivity to enzymatic digestion of DNAses, and by treating control and MA cells with DNAse I, we found that MA cells showed a chromatin conformation more resistant to DNA digestion (Fig. 7C). Furthermore, as actin might control also the chromatin compaction [28], we next investigated whether actin polymerization might regulate the global chromatin conformation in MA cells. We found that latrunculin B treatment reverted the chromatin state of MA cells, showing a DNA digestion profile more similar to control cells (Fig. 7C, D, Supplementary Fig. S8D, E). Consistent with this point, we performed in situ DNA digestion and verified that control cells were more sensitive to DNA digestion (visualized as a reduction in the nuclear area by DNAse activity) than MA cells (Supplementary Fig. S8F, G). To further characterize the global chromatin state of ORM cells, we performed a similar DNAse digestion assay and found that these cells presented a highly sensitive chromatin to DNA digestion, suggesting a more relaxed-chromatin conformation than control cells (Fig. 7E, F). Altogether, our results indicate that the nuclear response to osmotic shock and the chromatin compaction might be altered in cells that underwent confined migration.

### Confined migration promotes changes in the biomechanical landscape of the nucleus

**Fig. 8:**
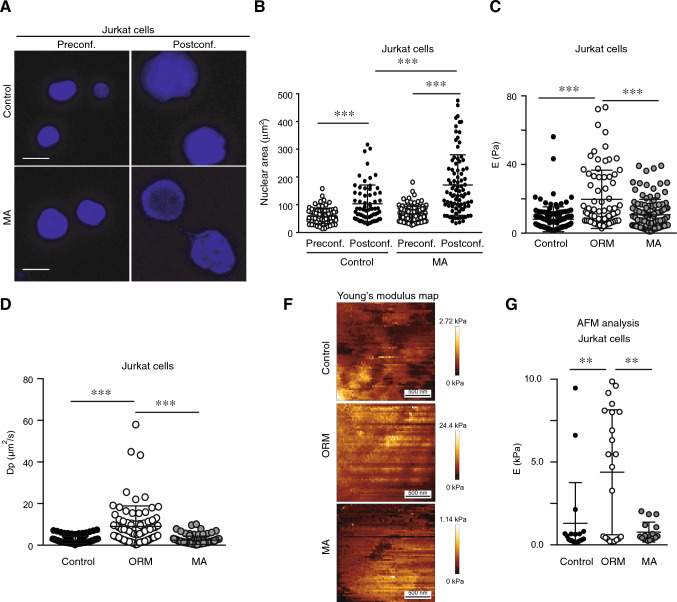
The nucleus alters its biomechanical behavior and compression in response to confined migration.** A** Isolated nuclei from control and MA Jurkat cells were stained with DAPI and seeded on poly-L-lysine-coated coverslips. Confocal sections of the nuclei were taken before (Preconf.) and after (Postconf.) confinement. Bar 10 μm. **B** Graph shows the nuclear area from cells in (**A**). Mean n = 66–89 isolated nuclei ± SD (3 replicates). **C**, **D **Isolated nuclei from control and MA Jurkat cells were seeded on polylysine-coated glasses and subject to indentation by optical tweezers. Graphs show the values of the stiffness (**E**) and the poroelastic diffusivity coefficient (Dp) calculated based on the nuclear indentation data for nuclei of control, ORM and MA cells. Mean n = 76–122 isolated nuclei ± SD (3 replicates). **E** Isolated nuclei from control, ORM, and MA cells were seeded on polylysine-coated dishes and imaged by AFM (Atomic Force Microscopy). PeakForce Tapping image of a representative nucleus for each condition captured with a maximum indentation force of 0.3 nN. Bar 500 nm. **F** Graph shows the Young's modulus values for isolated nuclei from control, ORM, and MA cells. Each point corresponds to the average value for a nucleus, calculated from at least 4000 force curves. Mean n = 17–22 isolated nuclei (4 replicates) ± SD. (***) P < 0.001.

As we described changes in lamin B1 and the chromatin state, both of which contribute to the mechanical properties and size of the nucleus [37], we exerted mechanical pressure forces onto isolated nuclei from control and MA cells and observed that the nuclear area of MA cells increased 2.5-fold under external pressure, whilst control cells showed only 1.77-fold (Fig. 8A, B, Supplementary Fig. S9A, and S9B). This indicates that MA cells showed a higher nuclear deformability compared to control cells. Furthermore, we observed a similar behavior upon mechanical compression for isolated nuclei from ORM cells that increased their nuclear deformability compared to control cells (Supplementary Fig. S9C). To assess the local mechanical response of isolated nuclei from control and migrating cells, we employed an approach based on the poroelastic behavior of the nucleus when subjected to chromatin deepening indentations using optical tweezers. By indenting at increasing depths across the nuclear periphery (~ 50–1200 nm), we obtained force-relaxation curves to estimate the stiffness (*E*), and the poroelastic diffusion coefficient (*D*_p_), both assumed inhomogeneous under exploring nuclear deepness. Whereas the former measures the local rigidity of the chromatin as a function of its molecular compaction, the second is a dynamic magnitude that accounts for the ability of water to permeate the nuclear porous structure upon compression. The results obtained for isolated nuclei from control, ORM, and MA cells revealed that ORM cells display a significant increase of both E and D_p_. These alterations of the nuclear mechanical properties were not detected in MA cells, whose response to optical tweezer indentation was similar to that observed for control cells (Fig. 8C, D). To further validate this observation, we performed atomic force microscopy (AFM) indentation of isolated nuclei from control, ORM, and MA cells and calculated the mean stiffness for the three populations. Our results revealed that isolated nuclei from ORM cells showed a heterogeneous population with an increased average value of E than their counterparts from control or MA cells (Fig. 8E and Supplementary Fig. S9D). Together, our results demonstrate that cells might modify the nuclear deformability upon mechanical forces and its biophysical landscape depending on transient or persistent migration.

## Discussion

In this work, we pointed out a connection between cell migration and permanent changes in the nucleus that lead to functional consequences in cell biology. The nucleus is a mechanosensory organelle that has to alter its mechanical and morphological characteristics for multiple cellular functions, including migration, development, and tumor invasiveness [38, 39]. To undergo migration, cells have to sense their physiological environment to control their integrity and functions [40, 41]. This is particularly important as extracellular migratory and mechanical signals regulate the adaptability of cells, their stemness, differentiation and genomic instability, and are linked to many human diseases [42–44].

Despite previous evidence showing that cell migration through confined spaces promotes nuclear ruptures, protein redistribution, and DNA damage in nuclear alterations [25, 45, 46], their long-term consequences remain still unclear. In contrast to the one-round of cell migration through 3 μm of pore size (where transient nuclear changes and lamin B1 distribution recovered their normal phenotype after 24 h), our persistent cell migration approach shows that MA cells adapted to three repeated rounds of cell migration by altering their nuclear morphology and lamin B1 distribution in a permanent manner. This transition highlights how cells might respond depending on the migratory stimulus and whether this is occasional or repeated. Furthermore, the aberrant redistribution of lamin B1 and other nuclear envelope components suggests that ORM and MA cells showed more nuclear lobs than control cells. A similar approach to study stable nuclear changes has been used in other cell types [19, 25, 47]; nonetheless, most of these changes were focused on lamin A and genomic alterations [47, 48], rather than on other nuclear components and the biomechanical aspect of these phenomena. Lamin A, and not lamin B, is the major contributor to the mechanical response of the nucleus [49]; and recently changes in nuclear adaptation depending on LAP1 and lamin A has been described, whilst the silencing of lamin B does not alter LAP1 adaptation [50]. Our observations related to lamin B1 redistribution differ from previous reports and might be of particular interest for hematological malignancies and immune cells, where lamin A expression is more restricted [20].

It has been reported the impact of cell confinement in stem cell reprogramming and cell rejuvenation [51–53]. Likewise, a broad range of changes related to transcription, chromatin compaction, and metastatic progression has been described upon mechanical constraints; nonetheless, these changes diverge depending on the mechanical stress in microfluidic devices, 3D conditions or specific cell culture confinement [54]. Our results suggest that confined migration promotes transcriptional changes in ORM and MA cells. Interestingly, minimal changes were observed in ORM cells compared to major transcriptional changes found in MA cells. Furthermore, only 10 altered genes were shared between ORM and MA cells, which might highlight how transient or persistent migration might have different impact on the genomic state of migrating cells. We identified changes in MA cells related to several biological processes, such as cell division and proliferation, in agreement with previous studies showing how biophysical stimuli control cell fate and differentiation [55, 56]. Indeed, there are evidences showing that mechanical stress regulates cell cycle progression and genomic instability that leads hyperploidy [57, 58]. Although we did not see significant changes in cell proliferation, we cannot discard that under physiological conditions or external stimuli, these changes might have potential implications in promoting cell state alterations and genomic instability and heterogeneity in cancer cells. In line with this idea, we observed that MA cells were more resistant to arrest in the G2/M phase compared to control cells when we forced their blocking with nocodazole.

This genomic instability observed in migrating cells might be linked not only to the cell cycle but also to DNA damage response, as recent studies have described the localization and upregulation of several markers of DNA damage in migratory cells under high nuclear deformation [59]. So far, nuclear lamins and deformability seemed to be involved in cell growth, DNA damage response, and cellular stress [60]. MA cells showed high basal levels of γH2AX and p-ATM but resisted apoptosis better than control cells. This chemoresistance to antimetabolites might be due to differences in cell proliferation or the metabolic activity of tumor cells [61]. Interestingly, our data indicated a downregulation in MA cells of genes related to unfolded protein response and endoplasmic reticulum stress, fundamental in chemoresistance [62]. While this is a simple model to induce apoptosis, a plausible explanation is that under specific circumstances, aberrant nuclear changes and high basal levels of DNA damage markers would be protective for MA cells against specific conventional therapies. This agrees with the idea that the steady state of γH2AX is critical for cell cycle regulation and the maintenance of genomic integrity [63, 64].

Although we observed increased chemotactic response of MA cells during migration, we found defective colonization of MA cells into different organs in vivo. The discrepancy between chemotactic response and cell homing into tissues might be due to the multistep biology of cell migration, as many different proteins and factors contribute to effective cell migration. For instance, an increment in the cell adhesion arrest might reduce the ability of cells to extravasate, and we have observed that MA cells increased the levels of β1 integrin. Moreover, when we attached cells on a 2D surface, we identified that ORM and MA cells showed differences in their cell spreading area compared to control cells. This suggests that other molecular pathways could also be regulating the migration strategy used by MA cells, which might become less invasive than control cells in vivo. Another plausible explanation of the defective cell migration in vivo of MA cells might be the migratory exhaustion of these cells, which would transit from a more effective movement into a more static condition. Together, our findings indicate that the potential adaptation of MA cells might have a dual effect on reducing their ability to reach other tissues, and on promoting other functions, such as cell survival and proliferation.

The cell skeleton, including actin polymerization and its associated proteins, is critical in many nuclear events, including chromatin compaction, and gene transcription [65, 66]. One exciting implication of our results is that actin depolymerization seemed to recapitulate the DNAse sensitivity of the chromatin in MA cells, in parallel to their lamin B1 redistribution at the nuclear periphery. Although it has been reported that myosin contractility transmits extracellular signals into mechanical changes [29], our data support the idea that the aberrant redistribution of lamin B1 observed in MA cells depends on actin polymerization rather than myosin activity. We also observed that latrunculin B treatment recovered the sensitivity of genomic DNA in MA cells, aligning with other results where actin homeostasis drugs alter the chromatin structure [67]. In fact, it has been reported that the actin cytoskeleton is critical for nuclear integrity and DNA repair in constricted migration [68]; and, in agreement with this, we observed that inhibiting actin polymerization also reduced the levels of γH2AX in MA cells.

It has been reported that cell migration through constrictions might alter genomic regions independently of transcriptional regulation [19]. As the nuclear changes observed in ORM and MA cells might have a direct role to modulate the biomechanical properties of the nucleus, and due to its role as a mechanosensor [69], we analyzed the mechanical features of the nucleus upon persistent and transient migration by combining nuclear swelling, nuclear compression, and indentation by optical tweezers and atomic force microscopy. Previous works show nuclear swelling and compression as good experimental approaches to study the mechanical response of the nucleus [70, 71]. Our experiments reveal that ORM and MA cells show significant differences from a mechanical point of view. On one hand, the global mechanical response of the isolated nuclei assessed by confinement assays showed that the nuclei of migrated cells (both ORM and MA) are more deformable. On the other hand, the study of the local mechanical response of the outer layer of the nuclei by optical tweezers and AFM (until ~ 1200 nm depth in optical tweezers and ~ 70 nm depth in AFM), exposed an increase of E and D_p_ in the nucleus of ORM cells, which was not observed in MA cells. In our experiments, we verified a distinct mechanical behavior of ORM and MA nuclei that might be due to the development of a sort of accommodation of the internal architecture of MA nuclei after three rounds of migration. This originates a more mechanically homogeneous MA nuclei population compared to that from ORM nuclei (this is reflected in the dispersion of E and Dp data). In addition, while MA cells presented more compacted chromatin than control cells, ORM cells showed a less dense chromatin state, which can account for the larger Dp values observed for this condition.

In conclusion, our findings highlight how migratory cells respond in structurally regulated ways to mechanical compression both in the short- and long-terms. We identify several nuclear and functional changes, which provide novel insights into alterations in the nucleus and functions of the cell. Finally, we demonstrate that altered nuclei showed quantitative differences and heterogeneity in their mechanical properties, given an integrative biophysical signature that might lead to broad implications for development, aging, cancer, and mechanobiological processes.

## Materials and methods

### Cell lines and culture

The human Jurkat (CVCL_0367) and CCRF-CEM (RRID: CVCL_0207) cell lines were from ATCC, American Type Culture Collection. Both cell lines were monthly tested for mycoplasm contamination and maintained in culture in RPMI 1640 medium with L-glutamine and 125 mM HEPES (Sigma Aldrich, St. Louis, MO, USA) and 10% fetal bovine serum, FBS (Sigma-Aldrich) and maintained in 5% CO_2_ and 37ºC.

One-round migration (ORM) cells were collected from the bottom chamber of Transwell inserts (Corning Costar, 6.5 mm diameter, 3 μm pore size) after 24 h. For recovery assays, collected ORM cells were used to perform experiments (fresh condition); or kept in culture conditions for an additional 24 h and then used to perform experiments (after 24 h condition). Migratory-altered (MA) cells were generated as follows: Jurkat or CCRF-CEM cells were allowed to migrate in transwells for 24 h; then, migrated cells were collected from the bottom chamber, counted and added again to the upper chamber of new inserts, under the same conditions. The same process was repeated 3 times. Finally, migrated cells from the bottom chamber were collected, expanded and kept in culture conditions as MA cells.

#### Immunofluorescence

Cells were cultured in suspension, treated or not with specific inhibitors, for 1 h at 37ºC. Cells were seeded onto poly-L-lysine-coated glass slides for 30 min. Then, cells were fixed with 4% paraformaldehyde (15 min) and permeabilized with 0.5% Tx-100 in PBS (5 min). Samples were blocked in 10% FBS and 0.1% Tx-100 in PBS for 1 h at RT, and then incubated with appropriated primary antibodies for an additional hour. After washing with PBS, samples were incubated with secondary antibodies (1 h at RT), stained with DAPI 1 µg/mL (10 min at RT), washed with PBS and H_2_O, and mounted. Images were acquired on an inverted DMi8 microscope (Leica) using an ACS-APO 63 × NA 1.30 glycerol immersion objective. Quantification and analysis of images were determined using ImageJ.

#### Electron microscopy

Cells were fixed for 1 h in 3% glutaraldehyde in PBS and then washed twice with PBS. Samples were post-fixed in 1% osmium tetroxide and 0.8% potassium ferricyanide for 1 h at 4ºC and washed with PBS prior to dehydration with an increasing gradient of ethanol (30%, 50%, 70%, 80%, 90% and 100%) of 10 min per step. Samples were embedded in LX112 resin and were polymerized for 48 h at 60ºC. 60–80 nm sections were placed in copper grids of 75 mesh and stained with 5% uranyl acetate for 30 min and lead citrate for 4 min. Samples were viewed in a JEOL 1230 TEM and images were taken with a CMOS TVIPS 16 Mpx camera.

#### Nuclear confinement

Cell nuclei were isolated by incubating cells in buffer A (10 mM HEPES (pH 7.9), 10 mM KCl, 1.5 mM MgCl_2_, 0.34 M sucrose, 10% glycerol, 1 mM dithiothreitol (DTT), 0.1% Tx100, and Roche protease inhibitor cocktail) for 5 min at 4 ºC and centrifuged for 5 min at 4 °C at 3500 rpm. Nuclei were resuspended in TKMC buffer (50 mM Tris pH [7.5], 25 mM KCl, 3 mM MgCl_2_, 3 mM CaCl_2_), stained with DAPI 1 µg/mL and sedimented onto poly-L-lysine coated plate for cell confinement analysis (4D cell). A glass slide with micropillars of 3 µm height was stuck on the silicone macropillar attached to the lid of the confiner. When the lid was closed, the pillars pushed the confining slides onto the culture substrate and confined the nuclei to 3 µm. Images of at least 20 nuclei were taken with a 63 × objective before and after the confinement by an inverted confocal microscope.

#### Flow cytometry

Isolated nuclei were resuspended in TKMC buffer and nuclear size was measured with a CytoFlex (Beckman Coulter). To analyze protein levels, cells were blocked with human IgG (1:1000; 30 min), incubated with specific primary antibodies of interest (1:1 to IgG), washed twice with PBS, followed by appropriated fluorochrome-conjugated secondary antibody for 30 min. For intracellular proteins quantification, cells were fixed with PFA 4% for 5 min at RT, permeabilized with PBS Tx-100 0.1% for 5 min, and stained as described above. To analyze the cell cycle progression, 3 × 105 cells were fixed in ice-cold 70% (v/v) ethanol overnight. Then, cells were centrifuged and the pellet was incubated in PBS with RNAse (100 μg/ml), 0.1% Triton X-100 and propidium iodide (50 μg/ml) for 30 min at RT. Cells were washed, resuspended in 600 μl of PBS-EDTA 2 mM and analyzed by flow cytometry. In some cases, cells were preincubated with nocozadole (100 ng/ml) for 16 h. Then, cells were fixed and stained as described above. Data analysis was performed using the FlowJo software and the cell cycle distribution determined by G1, S, and G2/M cell populations. Flow cytometry was performed with a FacSort. All data were analyzed using the Flow Jo software (Flow Jo LLC, Ashland, OR, USA).

#### Optical tweezers

Isolated cell nuclei resuspended in TKM buffer were mixed with polystyrene beads with a mean particle size of 3.0 μm (Sigma-Aldrich) at final concentrations of 10^7^ nuclei/ml and 0.005% (w/v), respectively. The optical tweezers device (SensoCell, Impetux Optics S.L.) is equipped with an ultra-stable single-frequency laser source (5 W, λ = 1064 nm) guided by acousto-optic deflectors and includes a direct force measurement platform capable of detecting the light scattered from the optical traps. The OT device is mounted on an inverted microscope (Eclipse Ti, Nikon) and a water immersion objective (Plan Apo VC 60XA/1.20 WI, Nikon) employed to focus the laser on the sample. A short-pass dichroic mirror transmits the bright-field illumination and reflects the IR trapping beam, and a short-pass filter was used to avoid IR laser radiation leaking. Sample bright-field imaging was captured by a CMOS camera (DCC1545M-GL, ThorLabs) and the optical traps were operated with LightAce software (Impetux Optics S.L.). For each indentation experiment, a volume of 40 μL of the sample was placed in a custom-made glass chamber. This chamber was mounted on the microscope and measurements were performed at 25 ºC. Only average-sized, round, symmetric nuclei with no alterations or major perturbations of their integrity were selected to perform the indentation routine. Indented nuclei got attached to the bottom surface of the glass chamber by unspecific interactions, and indenter beads were placed next to the nuclear envelope at axial positions and approximately 2 μm above the bottom surface of the chamber. Indenter beads had a nominal diameter of 3 µm, but the exact diameter of each bead used for indentation was measured by image analysis using the Fiji software. The indentation process consisted of pushing the nucleus radially by generating an oscillation of the indenting bead with a probing amplitude allowing to penetrate the nuclear chromatin but avoiding the outer nucleus membrane from breaking. The fixed parameters of the oscillation are the shape (squared), frequency (0.5 Hz, which is enough to permit a complete relaxation between consecutive cycles), and offset (100%). The amplitude was variable, and the routine was set to sweep the 0.6–1.6 μm range with steps of 0.05 or 0.10 μm. Data were acquired during 45 s for each amplitude step. For each indentation experiment, the elasticity constant of the trap was calculated by using a particle scan routine included in the LightAce software. We wrote a specific Matlab (MathWorks) script to analyze the data from the files obtained by the custom-made Labview indentation routine. This script analyzes each force–time curve for every indentation cycle and to calculate the stiffness from the indentation force values and the water diffusivity from the poroelastic relaxation times.

#### Statistics

Statistical analysis and comparisons were made with GraphPad Prism6. The numerical data are presented as mean ± SD. Differences between means were tested by Student's t test for two groups comparison. Where 3 or more groups were analyzed, one-way ANOVA was performed. P-values are indicated by asterisks ((*) P < 0.05; (**) P < 0.01; (***) P < 0.001).

## Consent for publication

All authors consent the manuscript for publication.

### Supplementary Information

Below is the link to the electronic supplementary material.

Supplementary file1 (DOCX 2617 KB)

Supplementary file2 (XLSX 46 KB)

Supplementary file3 (XLSX 18 KB)

Supplementary file4 (XLSX 14 KB)

Supplementary file5 (AVI 6618 KB)

Supplementary file6 (AVI 7097 KB)

Supplementary file7 (AVI 7929 KB)

Supplementary file8 (AVI 9180 KB)

Supplementary file9 (AVI 5563 KB)

Supplementary file10 (AVI 5843 KB)

Supplementary file11 (AVI 6408 KB)

Supplementary file12 (PDF 4468 KB)

## Data Availability

The accession numbers for the transcriptional microarray analyses are GSE214365 and GSE239463. All relevant data are available from the corresponding author upon reasonable request.
