# Supplementary material for: Mechanical stress confers nuclear and functional changes in derived leukemia cells from persistent confined migration
Source: Cell Mol Life Sci. 2023 Oct 6;80(11):316. doi: 10.1007/s00018-023-04968-5 (PMC10558412; doi:10.1007/s00018-023-04968-5)
Supplement: Supplementary file 1 — Supplementary file1 (DOCX 2617 KB) [file 18_2023_4968_MOESM1_ESM.docx]

**Mechanical stress confers nuclear and functional changes in derived leukemia cells from persistent confined migration.**

A. de Lope-Planelles, R. González-Novo, E. Madrazo, G. Peralta Carrero, M.P. Cruz Rodríguez, H. Zamora-Carreras, V. Torrano, H. López-Menéndez, P. Roda-Navarro, F. Monroy, J. Redondo-Muñoz.

**Supplementary Materials and Methods.**

**Supplementary Figures S1-S8.**

**Supplementary Tables S1 and S3.**

**Supplementary Movies S1-S7.**

**SUPPLEMENTARY MATERIALS AND METHODS**

| **Antibodies & reagents** | **Conc use** | **Host** | **Company** | **#Cat** |
| --- | --- | --- | --- | --- |
| Anti-lamin B1 | 1:100 | Mouse | Santa Cruz | 365962 |
| Anti-Emerin | 1:100 | Mouse | Santa Cruz | sc-2584 |
| Anti-Sun 2 | 1:100 | Mouse | Santa Cruz | Sc-377459 |
| Anti-pH2AX | 1:1000 | Rabbit | Cell Signaling | 2577 |
| Anti-EZH2 | 1:1000 | Rabbit | Cell Signaling | 5246 |
| Anti-SYK | 1:1000 | Rabbit | Cell Signaling | D3Z1E |
| Anti-PKC-β | 1:1000 | Rabbit | Thermo Fisher | PA5-13740 |
| Anti-WDR5 | 1:1000 | Rabbit | Bethyl | A302-430-A-M |
| Anti-beta 1 subunit | 1:100 | Mouse | Gift from Prof. Sánchez-Madrid | TS2/16 |
| Anti-pATM | 1:100 | Rabbit | Invitrogen | MA1-2020 |
| Anti-pH2AX | 1:100 | Mouse | Invitrogen | 14-9865-82 |
| Anti-phosphomyosin | 1:1000 | Mouse | Abcam | Ab277790 |
| Anti-β-actin | 1:100 | Rabbit | Sigma-Aldrich | SAB5600204 |
| CF647- anti-mouse IgG (H+L) | 1:100 | Donkey | Biotium | 20042 |
| CF594- anti-rabbit IgG (H+L) | 1:100 | Donkey | Biotium | 20152 |
| Anti-rabbit HRP- conjugated | 1:5000 | Goat | BioRad | 170-6515 |
| Anti-mouse HRP- conjugated | 1:5000 | Goat | BioRad | 170-6516 |
| Phalloidin |  |  | Invitrogen | A12379 |
| CellTrace™ CFSE | 1 µM | - | Thermo Scientific | C34554 |
| CellTrace™ Far Red | 1 µM | - | Thermo Scientific | C34564A |
| DAPI | 1:1000 | - | Sigma-Aldrich | 62248 |
| Poly-Lysine | - | - | Sigma-Aldrich | 25988-63-0 |
| DACO | - | - | Sigma-Aldrich | 10981 |
| Propidium iodide | 50 μg/ml |  | Sigma-Aldrich | P4864 |
| MTT | 1:10 | - | Sigma-Aldrich | CT01-5 |
| Methotrexate | 1 μM | - | Sigma-Aldrich | M9929 |
| Proteinase K | 1 μg/mL | - | Sigma-Aldrich | P1308 |
| RNAse A | 100 μg/ml | - | Sigma-Aldrich | R4875 |
| Roscovitine | 15 μM | - | Sigma-Aldrich | R7772 |
| Bleomycin | 40 μM | - | Sigma-Aldrich | B7216 |
| Chaetocin | 1 μM | - | Sigma-Aldrich | C9492 |
| Staurosporine | 50 nM | - | Sigma-Aldrich | 19-123-M |
| Enzastaurin | 2 nM | - | Sigma-Aldrich | SML0762 |
| Blebbistatin | 50 μM | - | Sigma-Aldrich | B0560 |
| Nocodazole | 100 ng/ml | - | Sigma-Aldrich | 487928 |
| Latrunculin B | 1 μg/mL | - | Enzo | BMC-T110_0001 |
| Jasplakinolide | 1 μg/mL | - | Enzo | ALX-350-275-C050 |
| DNAse I | 1U or 50U | - | Thermo Fisher | EN0521 |
| 35 mm glass-bottomed plates | - | - | Ibidi | 80136 |
| Phenol:chloroform:isoamyl alcohol | - | - | Panreac | A0889.0250 |

**Immunoblotting.** Cells were washed with cold PBS, lysed in sample buffer and then sonicated (15 s at 70 % amp) using a Microson XL2000 (Misonix). Protein samples were boiled and resolved by 7.5-15 % SDS-PAGE and transferred to nitrocellulose membranes (GE Healthcare Life Science). After electrophoresis, membranes were with 5% low fat milk in TBS-Tween (0.1 %) for 1h at RT and incubated overnight with primary antibodies (1:1000) at 4ºC with rotation. After washing with TBS-Tween 0.1%, membranes were incubated with HRP-labelled secondary antibodies (1:5000) for 1 hour at room temperature. Protein signal was developed using the enhanced chemiluminiscent detection method (Amersham) and analyzed in a ChemiDoc (Bio-Rad). For the stripping process, membranes were washed 3 times with stripping buffer (1.5% Glycine, 1% SDS and 1% NP-40 pH=2.2) for 1h each wash, before blocking and incubating with the proper primary antibodies as described above. Quantification and analysis of images was performed using ImageJ.

**Real-Time PCR (qPCR).** Total RNA was extracted using TRI reagent (Sigma) and genomic DNA was digested with 1U DNAse I, 2.5 mM MgCl2 (Promega) at 37ºC for 30 min. 5mM EDTA and 10 min at 65ºC was used to stop digestion. 1 μg of purified RNA was retrotranscribed using First Strand cDNA synthesis kit (Thermofisher) and cDNA concentrations were quantified using a NanoDrop ND-1000 Spectrophotometer (Fisher Scientific). Oligonucleotides for selected genes were designed according to the PrimerQuest Tool (IDT): *JAK2:* 5´-GCAACAGGAACAAGATGTGAAC-3´ and 5´-TTCCCTCCATTTCTGTCATCG-3´; *EZH2*: 5’-TTTCCAACACAAGTCATCCC-3’ and 5’-AACCCACATTCTTATCCCC-3; and *qTBP:* 5´-CGGCTGTTTAACTTCGCTTC-3´ and 5´-CACACGCCAAGAAACAGTGA-3´. Quantitative real-time PCR (qRT-PCR) was performed on a Roche LightCycler 96 following the manufacturer's instructions. Assays were made in triplicates and results normalized according to the expression levels of TBP. Melt curve analysis was performed at the end of PCR to confirm the presence of a single, specific product. The results were expressed using the ΔΔCt method for quantification.

**RNA microarray**. The mRNAs from cells were isolated using the NucleoSpin RNA kit (Macherey-Nagel), according to the manufacturer’s instructions. The RNA purity and concentration were determined by Nanodrop measurement, and 1 μg of RNA was used for microarray analysis by Human Gene Clariom S Assay (Thermo Fisher Scientific). Data were processed, normalized and log2 transformed by the UCM-Genomic CAI Unit. Analysis was performed using Transcriptome Analysis Console and Database for Annotation, Visualization and Integrated Discovery (DAVID) v6.8. Microarray data set used in this study is deposited at GEO (accession numbers #GSE214365 and #GSE239463).

**Osmotic stress**. Isolated nuclei were resuspended in TKMC buffer and sedimented onto poly-L-lysine coated plates. Nuclei were incubated or not with 5 μM EDTA (swelling condition) or MgCl_2_ (shrinking condition) for 10 min. Then, nuclei were fixed, permeabilized, and stained for their visualization on the microscope. In some cases, cells were pretreated with chaetocin (1 μM) for 1 h before nuclear isolation. Quantification and analysis of images were determined using ImageJ software.

**Transwell invasion**. Chemotaxis experiments were carried out using Transwell inserts (Corning Costar, 6.5 mm diameter, 3 μm or 5 μm pore size). 100 μL of serum-free RPMI containing 2x10^5^ ALL cells under specific conditions were added to the upper chamber of the insert, and 600 μL of RPMI supplemented with serum to the bottom chamber. After 24 h, migrated cells were collected from the bottom chamber and counted to calculate migration index.

**Cell penetration assay**. A 100 µl collagen matrix was reconstituted at 1.7 mg/mL in RPMI, neutralized with 7.5 % NaHCO_3_ and 25 mM HEPES inside Transwell inserts (0.4 µm, Costar). After 1 h at 37 ºC, 100 µL of serum-free RPMI containing 3x10^5^ cells was added on the top of it. RPMI medium with 10% of FBS was added to the bottom chamber of the Transwell as chemoatractant. After 24 h, invading cells were fixed with 4% PFA for 1 h, permeabilized with 0.5% Triton-X-100 in PBS for 30 min and stained with propidium iodide. Invading cells were imaged with a sCMOS Orca-Flash 4.0LT camera (Hamamatsu) coupled 5 to an inverted DMi8 microscope (Leica), capturing serial z- stacks every 10 µm with a 10× objective (dry ACS APO 10x/NA 0.3).

***In-vivo* invasiveness**. NOD-SCID-Il2rg-/- (NSG) mice (Mus musculus), were bred and maintained at the Servicio del Animalario del Centro de Investigaciones Biológicas Margarita Salas (CIB-CSIC) with number 28079-21A. All mice were used following guidelines issued by the European and Spanish legislations for laboratory animal care. 5x10^6^ cells were labeled with Far Red Cell Tracker (1 µM, control cells) and CFSE (1 µM, MA cells) for 30 min. Then, cell populations were mixed and intravenously (IV) administered to 15 weeks-old non-conditioned NSG mice. Sacrifice was performed 24 hours after injection. Bone marrow from femurs, the spleen and the liver were extracted and processed through mechanical disaggregation. The resulting tissues and the peripheral blood were processed with RBC lysis buffer (Thermo Scientific), filtered with a 100 μm strainer (ClearLine), and analyzed by flow citometry (FACSCantoTM II, Becton Dickinson).

**MTT proliferation assay.** 1x10^5^ cells were seeded into microplates in 100 μl culture medium for 24 or 48 h. Then, 10 μl of the MTT labeling reagent was added, and the microplate was kept for 4 h at 37°C in a humidified atmosphere. MTT was solubilized using 100 µL of isopropyl alcohol and 20 µL of PBS-3% SDS. Absorbance was measured at 560 nm (Varioskan, Thermo Fisher).

**BrdU cell proliferation assay**. 2x10^4^ cells per condition were collected and then processed with the BrdU cell Proliferation Assay Kit (Cell Signaling) following the manufacturer’s instructions. Briefly, after the BrdU addition the cells were incubated for either 4h or 18h at 37ºC to allow them to replicate. Then the cells were fixed and stained, first with a primary antibody for 1h at RT and then with an HRP-conjugated secondary antibody for 30 min at RT. Finally, HRP substrate TMB was added to develop color, and the absorbance was measured at 450 nm (Varioskan, Thermo Fisher).

**Comet assay.** To detect small amounts of DNA damage, including single and double-stranded breaks, the comet assay kit (Abcam) was used following the manufacturer’s instructions. Briefly, 3x10^5^ control, ORM, and MA cells were embedded in agarose at a ratio of 1:10 in comet slides. In some cases, cells were preincubated with with 40 µM Bleomycin for 4 hours at 37°C before their addition into the agarose. The slides were immersed in 4°C lysis buffer for 90 min and later in freshly prepared alkaline unwinding solution for 90 min at 4°C. The slides were then placed in an electrophoresis slide tray with the alkaline solution and electrophoresis was performed (18 V for 30 min). The cells in agarose were washed in distilled water, fixed in 70% cold ethanol, dried and stained with 0.1 µg/mL SYBR-Gold for 30 min at room temperature and then viewed. The tail moment (TM=tail length x % of DNA in the tail) was analyzed by the CometScore software (TriTek).

**Cell viability.** Cells were treated with or without methotrexate (0.5-2 μM) for 24h, collected, washed in PBS and stained using the Annexin V-FITC Apoptosis Detection kit (Immunostep) according to manufacturer’s instructions. The number of living cells was determined by Flow Cytometry, and data analysis was performed using the software FlowJo.

**DNAse I-sensitivity assay.** Cells were resuspended in Lysis buffer (Tris-HCl 10 mM [pH 7.5], sucrose 300 mM, NaCl 150 mM, MgCl_2_ 5 mM, NP-40 0.5%, DTT 0.5 μM) with protease inhibitors. Then, lysates were digested with 1 U of DNAse I diluted in DNAse buffer (Tris-HCl 10 mM [pH 7.5], MgCl_2_ 2.5 mM, CaCl_2_ 0.5 mM) for 8 or 15 min at 25°C. Reactions were stopped by adding STOP buffer (Tris-HCl 10 mM [pH 8], EDTA 5 mM, NaCl 200 mM, SDS 0.2%), and samples were subsequently incubated with RNAseA for 30 min at 37°C and proteinase K 60 min at 52°C. Digested DNA was washed with phenol/chloroform and precipitated with 2.5 volumes of ethanol and 3 M sodium acetate at −80°C overnight. DNA pellet was dissolved in water and resolved in 1.5% agarose gel.

***In situ* DNAse assay**. Cells were seeded onto polylysine-coated glasses for 15 min at 37ºC and treated with CSK buffer (PIPES/KOH 10mM, NaCl 100 mM, sucrose 300 mM, MgCl_2_ 1mM) for additional 5 min at RT. Then, cells were incubated with DNAse I (50 U/ml) for 20 min. Remaining nuclei were clarified and stained with CSK buffer plus 125 mM (NH_4_)_2_SO_4_ and Hoechst 33342 for 5 min at RT. Then, nuclei were fixed with methanol at −20°C for 5 min, washed with CSK buffer and mounted. Images were acquired on an inverted DMi8 microscope (Leica) using an ACS-APO 63x NA 1.30 glycerol immersion objective. Quantification and analysis of images were determined using ImageJ.

**Atomic Force Microscopy (AFM).** AFM measurements were performed with a Nanowizard V (Bruker) mounted on an inverted microscope (Nikon Eclipse Ti-U) connected to a 1/1.2" IDS 3060 USB3.0 camera. 1x10^6^ isolated nuclei from control, ORM, and MA cells were seeded on coverslips (25 mm diameter, 1.5 mm thickness) coated with 100 µg/mL polylysine. Imaging was performed in filtered PBS at room temperature. Pre-calibrated Silicon Tip – Nitride cantilevers (PFQNM-LC-V2 Bruker) were used with a 70 nm tip radius. QI mode for nuclei in liquid with a maximum indentation force of 0.3 nN was used in a central area of the nucleus (64x64 pixels, scan size of 2 µm). Indentation curves were fitted within JPK Data Processing (Bruker).

**SUPPLEMENTARY FIGURES**

**
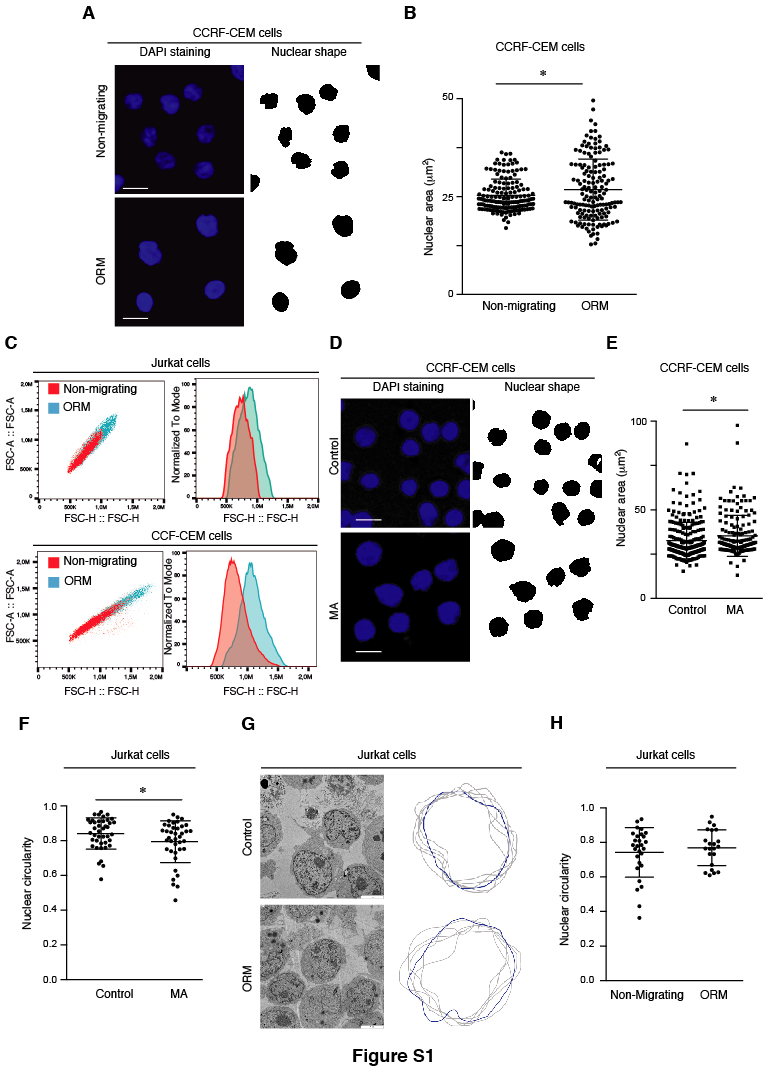
**

**Fig. S1. (A)** CCRF-CEM cells were allowed to migrate across 3 μm Transwell inserts for 24h. Non-migrating and one-round migrated (ORM) cells were collected from the upper and bottom chambers, respectively, sedimented on polylysine-coated glasses, fixed and stained with DAPI. Bar 10 μm. **(B)** Graph shows changes in the nuclear area of CCRF-CEM cells upon one round of migration through constrictions. Mean n= 157 cells ± SD (3 replicates). **(C)** Isolated nuclei from non-migrating and ORM cells were stained with DAPI, and their morphology and size were determined by flow cytometry. Graphs show the size of nuclei according to the FSC parameter. **(D)** Control and MA CCRF-CEM cells were sedimented on polylysine-coated glasses, fixed and stained with DAPI. Right panels indicate in black the area of the nuclei. Bar 10 μm. **(E)** Graph shows changes in the nuclear area of control and MA cells. Mean n= 161-258 cells ± SD (3 replicates). **(F)** Graph shows changes in the nuclear circularity of control and MA Jurkat cells quantified from images obtained in thin section electron microscopy. Mean n=39-41 cells ± SD. **(G)** Non-migrating and ORM Jurkat cells were collected and processed for thin section electron microscopy to visualize the nuclear morphology. Plots show changes in the nuclear circularity of non-migrating and ORM Jurkat cells (n=6 representative cells). **(H)** Graph shows changes in the nuclear circularity of non-migrating and ORM Jurkat cells quantified from images obtained in thin section electron microscopy. Mean n=22-28 cells ± SD.

**
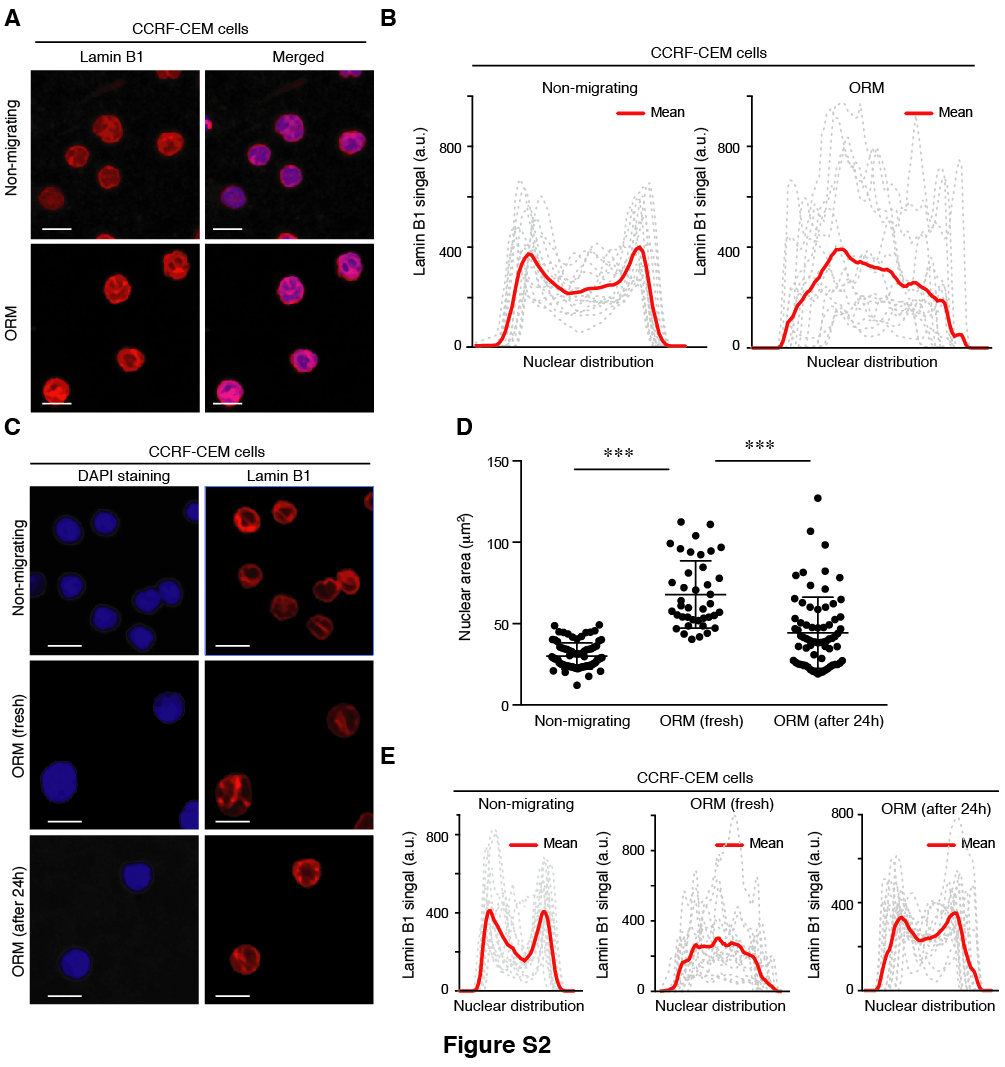
**

**Fig. S2. (A)** Non-migrating and ORM CCRF-CEM cells were seeded on polylysine-coated glasses and stained with DAPI (blue) and anti-lamin B1 antibody (red) for their analysis by confocal microscopy. Bar 10 μm. **(B)** Line plots show the signal profile of lamin B1 from 15 representative nuclei. Red line indicates the mean intensity of the profiles analyzed. **(C)** Non-migrating, fresh ORM, and ORM CCRF-CEM cells collected and cultured in suspension for an additional 24 h were seeded on polylysine-coated glasses and analyzed by confocal microscopy. Bar 10 μm. **(D)** Graph shows changes in the nuclear area of the cells from (C). Mean n=40-71 cells ± SD (2 replicates). **(E)** Line plots show the profiles based on lamin B1 intensity across 15 representative nuclei of the cells from (C). Red line indicates the mean intensity of the profiles analyzed.


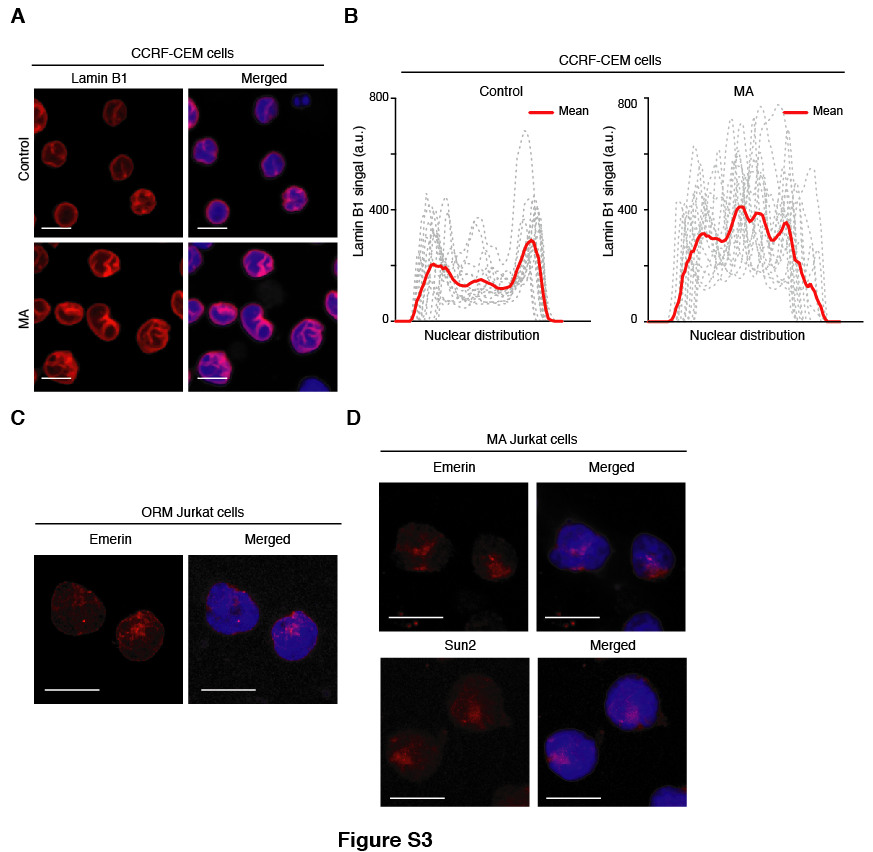


**Fig. S3. (A)** Control and MA CCRF-CEM cells were seeded on polylysine-coated glasses and stained with DAPI (blue) and lamin B1 (red). Bar 10 μm. **(B)** Line plots show the signal profile of lamin B1 from 15 representative nuclei. Red line indicates the mean intensity of the profiles analyzed. **(C)** ORM Jurkat cells were seeded on polylysine-coated glasses and stained with DAPI (blue) and emerin (red). Bar 10 μm. **(D)** MA Jurkat cells were seeded on polylysine-coated glasses and stained with DAPI (blue) and the specific nuclear envelope markers emerin and Sun2 (red). Bar 10 μm.


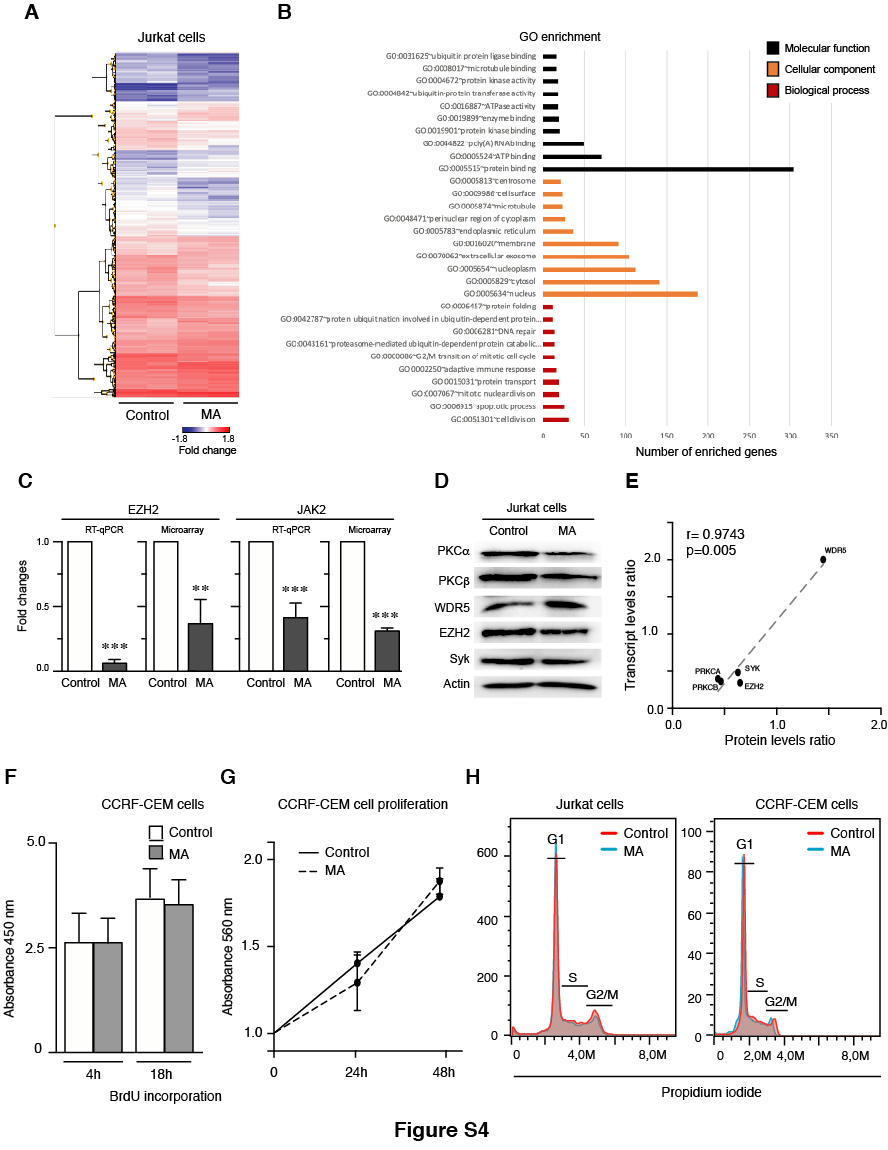


**Fig. S4. (A)** Control and MA Jurkat cells were lysed, and transcriptional changes determined by mRNA expression microarray. Heat map shows the relative gene expression patterns of control and MA cells. **(B)** Graph shows the top gene ontology (GO) enrichment results from the microarray analysis. **(C)** Graph shows the validation of microarray by qPCR. The expression of EZH2 and JAK2 was detected in control and MA cells by qPCR (3 replicates) and the microarray data (2 replicates). Error bars indicate standard deviations. **(D)** Representative immunoblots for PKCα, PKCβ, syk, EZH2 and actin in the whole cell lysates of control and MA Jurkat cells. **(E)** Plot shows the transcript-protein level pairs from 5 randomly selected proteins from the transcriptional analysis by microarray. Gene names, Pearson correlation analysis (r) and P value are indicated. **(F)** Control (dark line) and MA (dashed line) CCRF-CEM cells were cultured at indicated times and cell proliferation was quantified by MTT assay. Mean n = 3 replicates ± SD. **(G)** Control and MA CCRF-CEM cells were incubated with BrdU for 4 and 18 h. Then, cells were fixed and BrdU incorporation was quantified. Mean n = 6 replicates ± SD. **(H)** Control (red) and MA (blue) Jurkat and CCRF-CEM cells were fixed, permeabilized and stained with propidium iodide. Then, cell cycle progression was analyzed by flow cytometry. Graph shows the G1, S and G2/M phases according to DNA content.


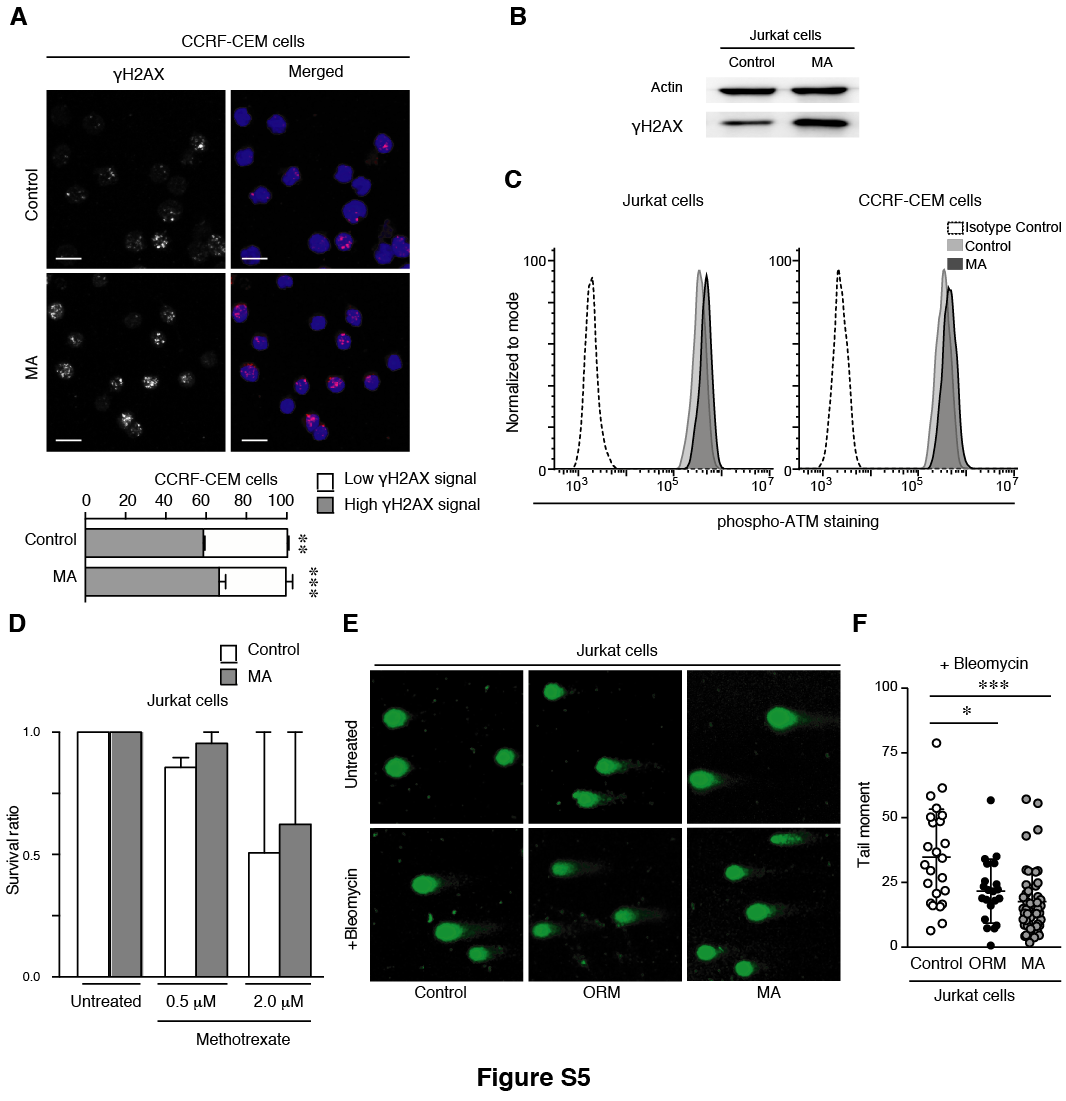


**Fig. S5. (A)** Control and MA CCRF-CEM cells were seeded on polylysine-coated glasses, stained, and analyzed by confocal microscopy. Bar 10 μm. Graph shows the percentage of control and MA CCRF-CEM cells with more than 2 visible foci for γH2AX. Mean n = 146-171 cells ± SD (3 replicates). **(B)** Control and MA Jurkat cells were lysed and the levels of γH2AX were resolved by westernblotting. Actin was used as internal loading control. **(C)** Control and MA cells were fixed, permeabilized and the levels of phospho-ATM were determined by flow cytometry. **(D)** Control and MA Jurkat cells were cultured in the presence or not of methotrexate (0.5 and 2 μM) for 24 h. Then, cells were collected and stained with annexin V-FITC and propidium iodide for their flow cytometry analysis. **(E)** Control, ORM, and MA Jurkat cells were cultured in the presence or not of bleomycin (40 µM) for 4 h. Then, cells were embedded in agarose, lysed and the DNA fluorescence was visualized by alkaline comet assay **(F)** Graph shows the tail moment analysis of the comet assay in (E). Mean n = 33-88 cells ± SD (2 replicates).


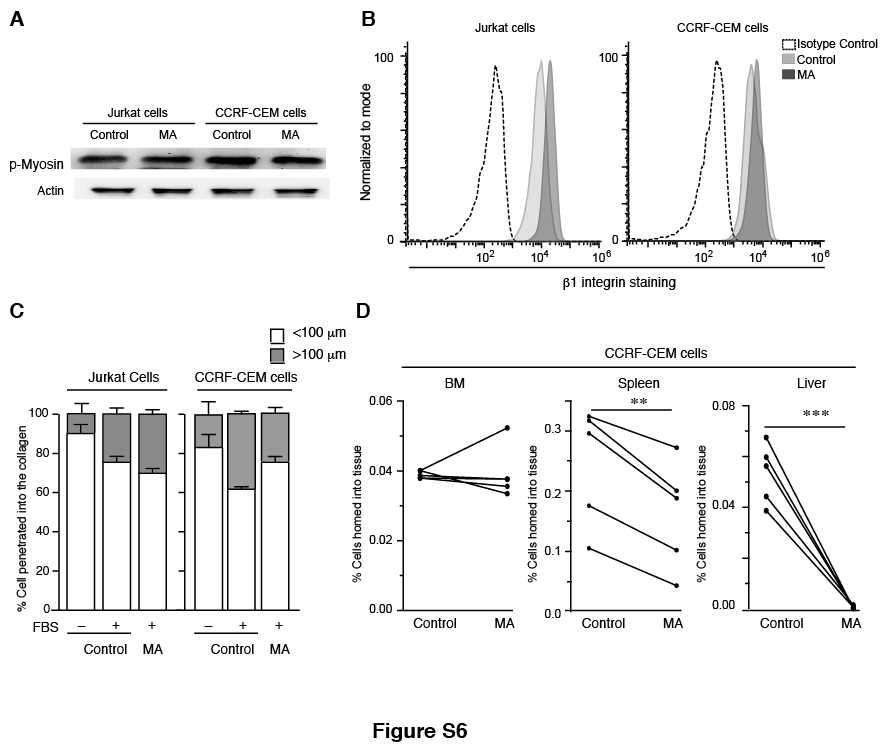


**Fig. S6. (A)** Control and MA Jurkat and CCRF-CEM cells were lysed and the levels of phospho-myosin were resolved by western blotting. Actin was used as internal loading control. **(B)** Graphs show then expression levels of the integrin subunit β1 at the cell surface of control and MA Jurkat and CCRF-CEM cells. **(C)** Control and MA Jurkat and CCRF-CEM cells were seeded on the top of a collagen matrix and allowed to penetrate into the collagen in response to serum (FBS, fetal bovine serum) for 24 h. Cells were fixed, stained with propidium iodide and serial confocal sections were captured. Graph shows the percentage of cells invading deeper than 100 μm. Mean n=3 replicates ± SD. **(D)** Control (Cell Tracker Far Red+) and MA (CFSE+) CCRF-CEM cells were mixed 1:1 and injected into the tail vein of 5 NSG mice. After 24 h, mice were sacrificed, and labeled cells from spleen, liver and bone marrow were collected and determined by flow cytometry. Graph shows the percentage of control and the MA cells analyzed in each animal. Mean n = 5.


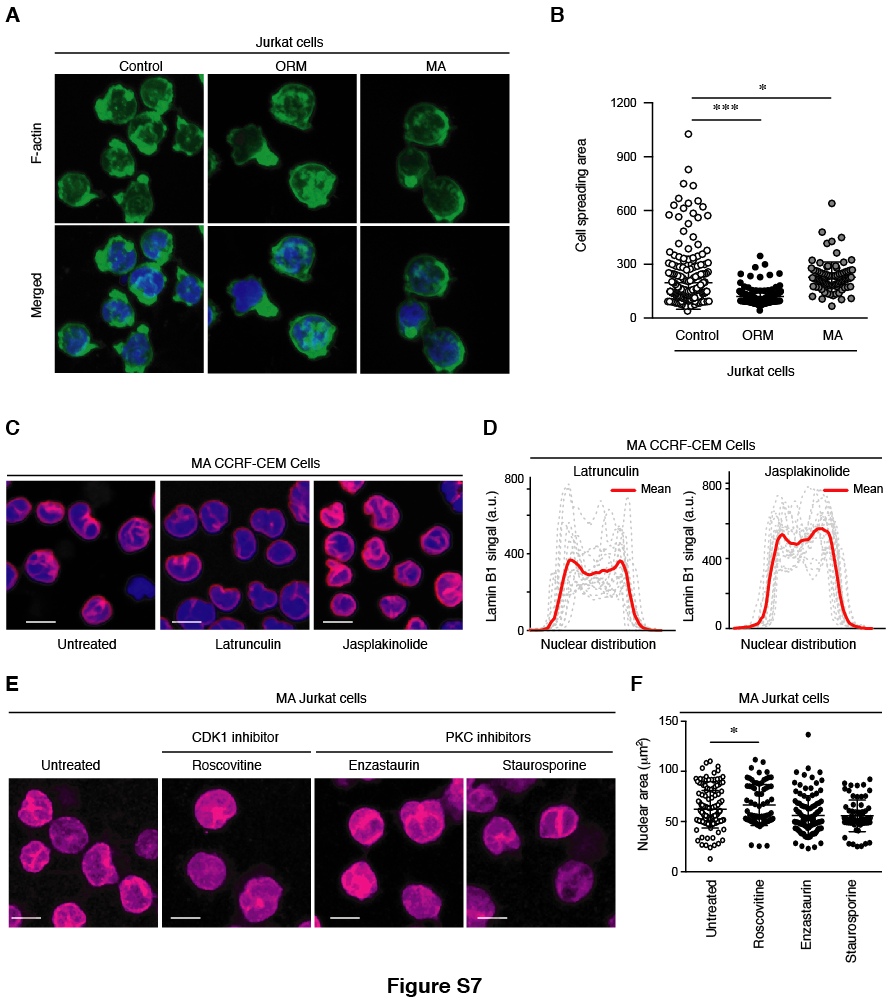


**Fig. S7. (A)** Control, ORM, and MA Jurkat cells were cultured on polylysine-coated glasses, fixed, permeabilized and stained for the indicated molecules. Bar 10 μm. **(B)** Graph shows the mean of cell spreading area of cells in (A). Mean n=89-246 cells ± SD (3 replicates). **(C)** MA CCRF-CEM cells were cultured in the presence or absence of latrunculin B (1 μg/mL) and japlakinolide (1 μg/mL) for 1 h at 37ºC. Then, cells were seeded on polylysine-coated glasses, fixed, permeabilized and stained with DAPI (blue) and anti-laminB1 (red) antibody. Bar 10 μm. **(D)** Line plots show the signal profile of lamin B1 from 15 representative nuclei. Red line indicates the mean intensity of the profiles analyzed. **(E)** Control and MA Jurkat cells were cultured in the presence or absence of roscovitine (15 μM, Cdk1 inhibitor), enzastaurin and staurosporine (50 nM, 2 nM, respectively, PKC inhibitors), for 30 min. Then, cells were fixed, permeabilized and stained with DAPI and anti-laminB1 antibody. Bar 10 μm. **(F)** Graph shows changes in the nuclear area of MA cells upon treatments in (C). Mean n=65-186 cells ± SD (3 replicates).


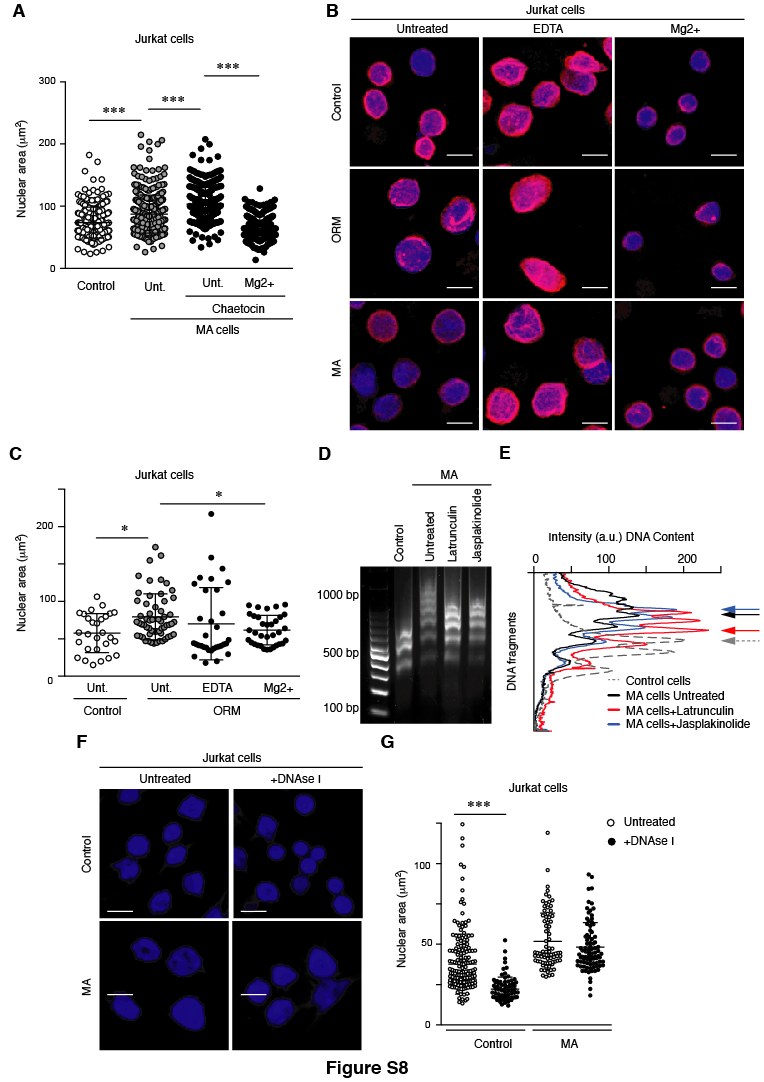


**Fig. S8. (A)** MA cells were preincubated with chaetocin (1 μM) for 1 h, then, nuclei from control and MA cells were isolated, sedimented on polylysine-coated glasses, and incubated under specific osmotic conditions induced by the addition of EDTA (swelling conditions) or MgCl_2_ (shrinking conditions). Then, nuclei were fixed, permeabilized, DAPI-stained and graph shows the nuclear area. Mean n = 206-360 isolated nuclei ± SD (3 replicates). **(B)** Isolated nuclei from control, ORM, and MA Jurkat cells were seeded on polylysine-coated glasses and incubated under specific osmotic conditions. Then, nuclei were fixed, permeabilized and stained with DAPI and an anti-lamin B1 antibody for their analysis by confocal microscopy. Bar 10 μm. **(C)** Graph shows the nuclear area of isolated nuclei of control and ORM Jurkat cells under osmotic stress conditions induced by the addition of EDTA (swelling conditions) or MgCl_2_ (shrinking conditions). Mean n = 11-81 isolated nuclei ± SD (2 replicates). **(D)** Control and MA Jurkat cells were incubated with indicated treatments, collected, and their DNA was digested with DNAse for 8 min. Then, DNA fragments were resolved in an agarose gel. **(E)** Graph shows the DNA degradation profile from control (dashed line) and MA Jurkat cells as in (D). Arrows indicate the maxima DNA peaks in each cell population. **(F)** Control and MA Jurkat cells were collected, and their DNA was digested with DNAse for 20 min. Then cells were stained with DAPI (blue), fixed, and analyzed by confocal microscopy. Bar 10 μm. **(G)** Graph shows changes in the nuclear area of control and MA Jurkat cells as in (G). Mean n=72-185 cells ± SD.


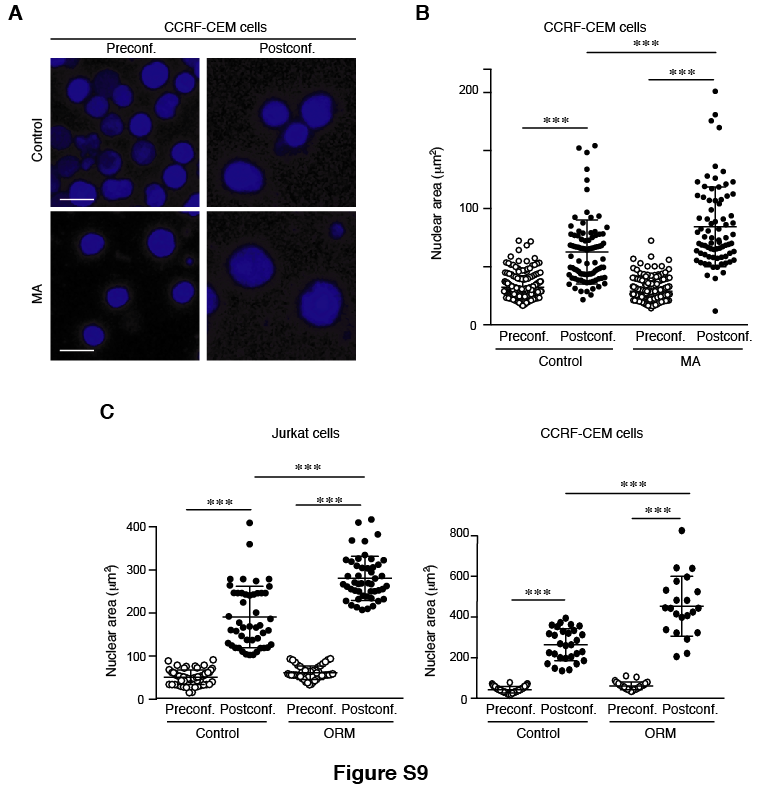


**Fig. S9. (A)** Isolated nuclei from control and MA CCRF-CEM cells were stained with DAPI and seeded on polylysine-coated glasses. Nuclear area was determined before (Preconf.) and after (Postconf.) confinement. Bar 10 μm. **(B)** Graph shows the nuclear deformability upon mechanical compression. Mean n = 78-88 isolated nuclei ± SD (3 replicates). **(C)** Graphs show the nuclear deformability upon mechanical compression of isolated nuclei from control and ORM Jurkat and CCRF-CEM cells. Nuclear area was determined before (Preconf.) and after (Postconf.) confinement. Bar 10 μm. Mean n = 29-63 nuclei ± SD (2 replicates).

**SUPPLEMENTARY TABLES**

**Supplementary Table S1**. Transcriptional changes of control and MA Jurkat cells by microarray analysis. |Fold Change| > 1.8 and P-value <0.05. n=2.

**Supplementary Table S2**. Transcriptional changes of control and ORM Jurkat cells by microarray analysis. |Fold Change| > 1.8 and P-value <0.05. n=2.

**Supplementary Table S3**. The table shows the terms from the microarray analysis with the highest significance according to the adjusted FDR p-value, ordered by pathway.

**SUPPLEMENTARY MOVIES**

**Supplementary Movies S1-S4**. 3D reconstructions from confocal sections of representative lamin B1-stained nuclei of non-migrating (S1), ORM (S2), control (S3) and MA (S4) Jurkat cells.

**Supplementary Movies S5.** 3D reconstructions from confocal sections of representative emerin-stained nucleus of ORM.

**Supplementary Movies S6, S7.** 3D reconstructions from confocal sections of representative nucleus of MA cells stained for emerin (S6) or Sun2 (S7).
