# Supplementary material for: Mechanical stress confers nuclear and functional changes in derived leukemia cells from persistent confined migration
Source: Cell Mol Life Sci. 2023 Oct 6;80(11):316. doi: 10.1007/s00018-023-04968-5 (PMC10558412; doi:10.1007/s00018-023-04968-5)
Supplement: Supplementary file 12 — Supplementary file12 (PDF 4468 KB) [file 18_2023_4968_MOESM12_ESM.pdf]

Persistent migration through narrow spaces confers permanent transcriptional and functional changes in migrating cells.

Full unedited gels for Figure S4D. Lane 1: control Jurkat cells. Lane 2: MA Jurkat cells.

PKCα protein expression.

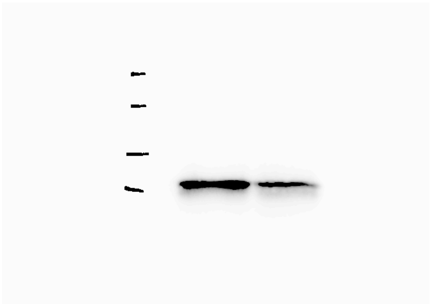

PKCβ protein expression

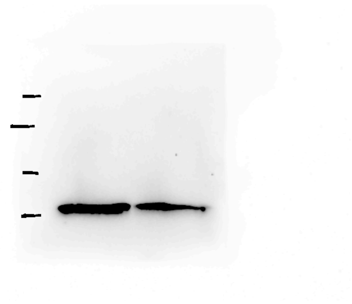

WDR5 protein expression.

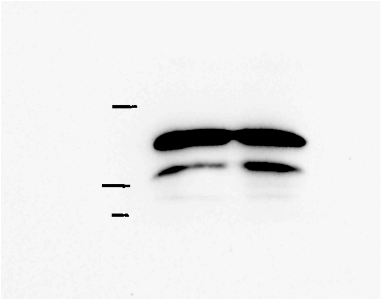

EZH2 protein expression.

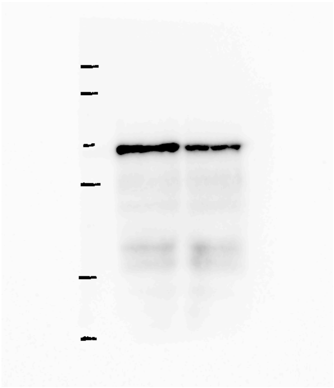

Syk protein expression.

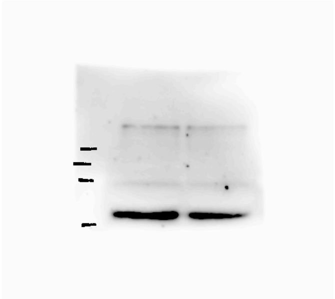

Actin protein expression.

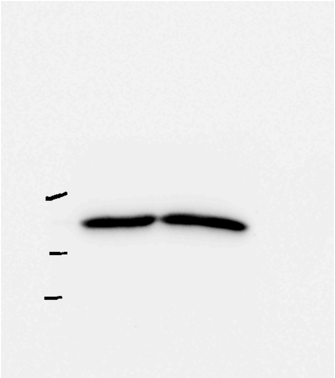

Full unedited gels for Figure S5B. Lane 1: control Jurkat cells. Lane 2: MA Jurkat cells.

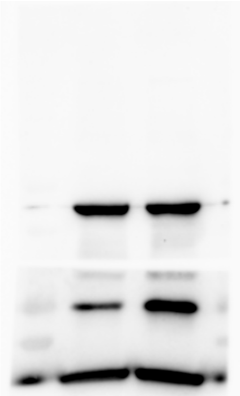

Actin protein expression.

Phospho H2AX.

Full unedited gels for Figure S6A. Lane 1: control Jurkat cells. Lane 2: MA Jurkat cells. Lane 3: control CCRF-CEM cells. Lane 2: MA CCRF-CEM cells.

Phospho myosin (MLC) protein expression.

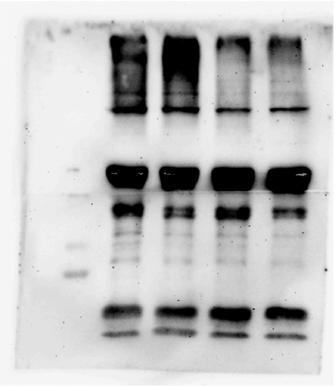

Actin protein expression.
